# Recent Advances in the Drugs and Glucose-Responsive Drug Delivery Systems for the Treatment of Diabetes: A Systematic Review

**DOI:** 10.3390/pharmaceutics16101343

**Published:** 2024-10-20

**Authors:** Junyu Liu, Xudong Yi, Jinrui Zhang, Yiman Yao, Pharkphoom Panichayupakaranant, Haixia Chen

**Affiliations:** 1 Tianjin Key Laboratory for Modern Drug Delivery and High-Efficiency, School of Pharmaceutical Science and Technology, Faculty of Medicine, Tianjin University, Tianjin 300072, China; 2 Phytomedicine and Pharmaceutical Biotechnology Excellence Center, Faculty of Pharmaceutical Sciences, Prince of Songkla University, Hat-Yai, Songkhla 90112, Thailand

**Keywords:** drugs on diabetes, glucose-responsive, drug delivery system, glucose oxidase, phenylboronic acid, Concanavalin A

## Abstract

Diabetes is a common chronic metabolic disease. Different types of drugs play important roles in controlling diabetes and its complications, but there are some limitations. The glucose-responsive drug delivery system is a novel technology with potential in diabetes treatment. It could automatically release drugs in response to changes in glucose levels in the body to maintain blood glucose within a normal range. The emergence of a glucose-sensitive drug delivery system provides a more intelligent and precise way to treat diabetes. The review is carried out according to the Preferred Reporting Items for Systematic Reviews (PRISMA 2020) guidelines This review focuses on the recent advances in the drugs and different systems of glucose-sensitive drug delivery, including glucose oxidase, phenylboronic acid, Concanavalin A, and other glucose-reactive systems. Furthermore, the glucose-responsive drug delivery system combined with the application applied in hydrogels, microneedles, and nanoparticles is also explored and summarized. The new platforms to sustain the release of anti-diabetic drugs could be desirable for patients. It could lead to increased adherence and glycemic outcomes for the detection and treatment of diabetes. Furthermore, given the limitations of glucose-responsive drug delivery systems, solutions and perspectives are proposed to help the understanding and application of these systems. This review will be helpful for drug discovery and treatment of diabetes from a new perspective.

## 1. Introduction

Diabetes mellitus is a metabolic disorder characterized by the pancreas leaking insulin or the insensitivity of the body to insulin. It is mainly divided into type 1 diabetes (T1D), type 2 diabetes (T2DM), gestational diabetes, and secondary diabetes [1]. T2DM, formerly known as noninsulin-dependent diabetes mellitus or grown-up starting diabetes, is the main type of diabetes. Usual signs contain common urination and may include gorge, tiredness, or soreness. Long-term problems of hyperglycemia include emotional illness and diabetic retinopathy. It could result in blindness, kidney failure, amputation, and diabetic ketoacidosis [2]. The chief medicines for diabetes are insulin and hypoglycemic drugs, which contain guanidine, sulfonylureas, thiazolidinedione, dipeptidyl peptidase-4 receptor inhibitors, sodium-dependent glucose transporters 2 receptor inhibitors, glucagon-like peptide-1 analogs and some natural products and traditional medicines [3]. These medications are designed to be hypoglycemic and reduce the risk of diabetes complications. Efficient management of diabetes often needs a mixture of medicine, lifestyle changes, and checking of blood glucose levels to stop serious and persistent problems. Since there is no cure for diabetes at present, the patient needs to take medication for their whole life. Medication research should seek new ways to reduce the pain of diabetes and improve the efficacy of hypoglycemic drugs.

The drug delivery system (DDS) is an example of the research into new dosage types in modern pharmacy and is the crystallization of the development of current discipline and technology. DDS has produced large developments in continuous-release, percutaneous, and marked drug delivery systems [4,5]. The glucose-responsive drug delivery system acts as an innovative move toward diabetes. It could release medication in response to changes in glucose position in the physique, hence continuing their lifeblood sugar position within a usual series. The system typically consists of two practical parts: a glucose faculty part (which discovers changes in glucose attention in the physique) and an insulin shop and set-free component (which is to blame for storing and later setting insulin free). Combining these components into one system allows for more precise control of the delivery of hypoglycemic drugs such as insulin. It can reduce the risk of hypoglycemia and enhance the overall management of diabetes. They could offer significant advantages over traditional insulin therapy, potentially changing the treatment landscape for diabetes [6]. In the existing literature, three kinds of glucose-sensitive materials have been considered most widely—glucose oxidases (GOx), phenylboronic acid (PBA), and Concanavalin A (Con A). All of these could interact with glucose in a particular way and cause changes in microenvironmental state or substance. At the same time, nanospheres, micelles, vesicles, hydrogels, and nuclei have been successfully designed as medication carriers for diabetes.

This review focuses on the recent advances in the drugs and different systems of glucose-sensitive drug delivery, containing GOx, PBA, Con A, and other glucose-reactive systems. Combined with hydrogels, microneedles, and others to maintain the release of medication, it could go in front to raise obedience and glycemic results for diagnosis and care of diabetes. Furthermore, given the advantages and limitations of glucose-responsive drug delivery systems, the review and perspectives are proposed to help learn it for diabetes treatment.

## 2. Methods

This article is inclusively based on the previously published literature.

### 2.1. Eligibility Criteria

This review summarizes the advances in drugs and glucose-responsive drug delivery systems for treating diabetes. The search included only review articles and patents in English, published in the past 10 years (2014–2023), and containing the selected keywords.

### 2.2. Information Sources

Information for the past 10 years was searched using the databases PubMed and Web of Science, and the review was carried out according to the Preferred Reporting Items for Systematic Reviews (PRISMA 2020) guidelines [7].

### 2.3. Search Strategy

The categories of glucose-responsive drug delivery systems for diabetes and main drugs for diabetes are listed according to the literature, and different categories of drug delivery systems were used as terms to retrieve related introductions (Table 1).

### 2.4. Study Selection and Data Collection Process

We used a PRISMA 2020 flow diagram to extract the most relevant data essential for synthesizing the results. Titles and abstracts were systematically screened and assessed by two independent reviewers. This review exclusively focused on results obtained from the Web of Science and PubMed databases. The title, abstract, author name, journal name, and year of publication of each study were exported to a Microsoft Excel spreadsheet, with duplicates removed. Subsequently, the aforementioned inclusion and exclusion criteria were applied to the full-text articles. Any discrepancies between the reviewers were addressed through discussion until a consensus was achieved.

## 3. Results and Discussion

### 3.1. Database Search and Included Studies

**Figure 1:**
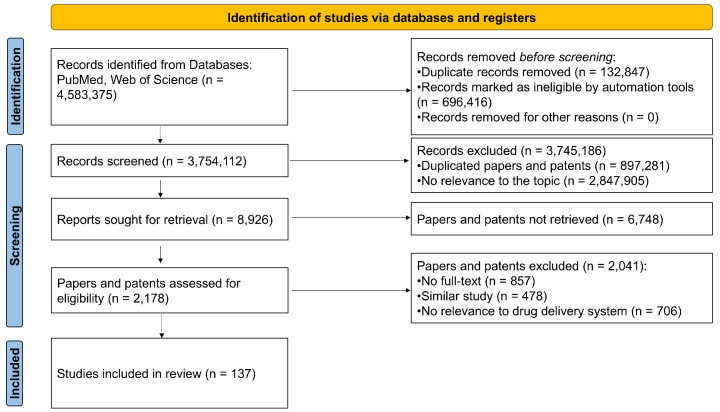
The flowchart of the selection process of literature and reports based on PRISMA.

The flowchart illustrating the literature retrieval and selection process for this review is presented in Figure 1. Two authors retrieved the research. In case of inconsistent answers, following the discussion results and deciding jointly, a total of 4,583,375 records were initially identified. Of these, 829,263 records were excluded for various reasons: book chapters, letters, news articles, conference papers, reports, etc. Following a database screening that eliminated duplicate entries and records not pertinent to the topic, 8926 studies were ultimately identified. Subsequent screening of titles and abstracts resulted in the retention of 2178 records due to their relevance and alignment with the scope of this review. The full texts of these papers were then thoroughly reviewed and assessed to ascertain their compliance with the criteria established for this scoping review. Ultimately, 137 studies were included in the final analysis. Table S1 presents the characteristics of the studies included in this systematic review.

### 3.2. The Advanced Progress of Drugs in the Treatment of Diabetes

The World Health Organization (WHO) recommends the utilization of HbA1c for the diagnosis of diabetes, with a diagnostic cut-off point established at HbA1c ≥ 6.5% [8]. According to the guidelines from the United States, Europe, China, Japan, and other regions, diabetes is classified into four categories based on etiological evidence: type 1 diabetes mellitus (T1D), type 2 diabetes mellitus (T2DM), gestational diabetes, and special types of diabetes [9,10]. The pharmacological management of hyperglycemia typically focuses on addressing two primary pathophysiological alterations that contribute to elevated blood glucose levels: insulin resistance and impaired insulin secretion. Oral hypoglycemic agents could be categorized based on their mechanisms of action. The primarily enhanced insulin secretion includes sulfonylureas, glinides, and dipeptidyl peptidase-4 inhibitors (DPP-4i). Other agents that lower blood glucose through alternative mechanisms encompass biguanides, thiazolidinediones (TZD), α-glucosidase inhibitors, and sodium–glucose cotransporter 2 inhibitors (SGLT2i) [11].

For T1D patients, the immune system erroneously targets and destroys the insulin-producing beta cells located in the pancreas, which leads to a complete deficiency of insulin. Consequently, patients with T1D require exogenous insulin therapy to effectively regulate blood glucose levels and manage their overall health [12]. Insulin therapy may also be employed for glycemic control in patients with T2DM, particularly as the disease progresses to more advanced stages or when complications arise [13]. Metformin is recognized as the first-line pharmacological agent. It serves as the foundational component in the treatment of hyperglycemia among T2DM patients. Other significant pharmacological agents may be combined with metformin. These include sulfonylureas, glinides, α-glucosidase inhibitors, thiazolidinediones, dipeptidyl peptidase-4 inhibitors (DPP-4i), sodium–glucose cotransporter-2 inhibitors (SGLT2i), glucagon-like peptide-1 receptor agonists (GLP-1RA), and insulin [14]. Metformin enhances glucose utilization by diminishing hepatic glucose production and improving insulin sensitivity in peripheral tissues [15]. However, it is primarily excreted via the kidneys. Metformin may accumulate to potentially harmful levels in patients with compromised renal function, thereby increasing the risk of adverse reactions. Despite these concerns, metformin remains the preferred treatment for T2DM globally [16]. Acarbose, glibenclamide, and liraglutide exhibit strong synergistic effects when combined with metformin. However, the development of new pharmacological agents continues. Tirzepatide is a long-acting dual GLP-1/GIP receptor agonist that is injected once a week. It was approved by the FDA in November 2023. It is indicated for chronic weight management in adults with overweight (BMI ≥ 27) or obesity (BMI ≥ 30) who have one or more weight-related comorbidities (such as hypertension, dyslipidemia, or T2DM). It has been demonstrated to have significant hypoglycemic and weight loss effects in diabetic patients. The 2023 American Diabetes Association (ADA) guidelines classify tripeptide as the most effective agent for both weight loss and glycemic control [17]. Insulin icodec is a basal insulin analog that employs fatty acid acylation technology to enhance its binding affinity to albumin. It could reduce receptor-mediated clearance in vivo and extend its half-life, which in turn prolongs its hypoglycemic effects. This agent was approved by the China Food and Drug Administration in May 2023 [18]. Orforglipron is recognized as the world’s first non-peptide oral GLP-1 receptor agonist. It exhibits resistance to enzymatic degradation and demonstrates greater stability within the body. Its oral administration is not constrained by food intake or hydration. In 2023, phase 2 clinical trials of Orforglipron revealed significant hypoglycemic and weight loss effects. Its overall safety profile is comparable to that of other GLP-1 receptor agonists. This led to its advancement into phase 3 clinical trials [19]. Additionally, Lantidra represents the first allogeneic islet cell therapy. It was derived from donor islet cells, which involves the injection of islet cells extracted from a deceased donor’s pancreas into a patient’s hepatic portal vein, accompanied by the administration of immunosuppressive agents to preserve islet cell viability. The FDA approved Lantidra for the treatment of diabetes on 28 June 2023 [20]. Nowadays, the development of new pharmacological agents continues. The treatment of diabetes must progress towards more sophisticated and precise drug delivery systems across all dimensions.

### 3.3. Classification of Glucose-Responsive Drug Delivery Systems

**Figure 2:**
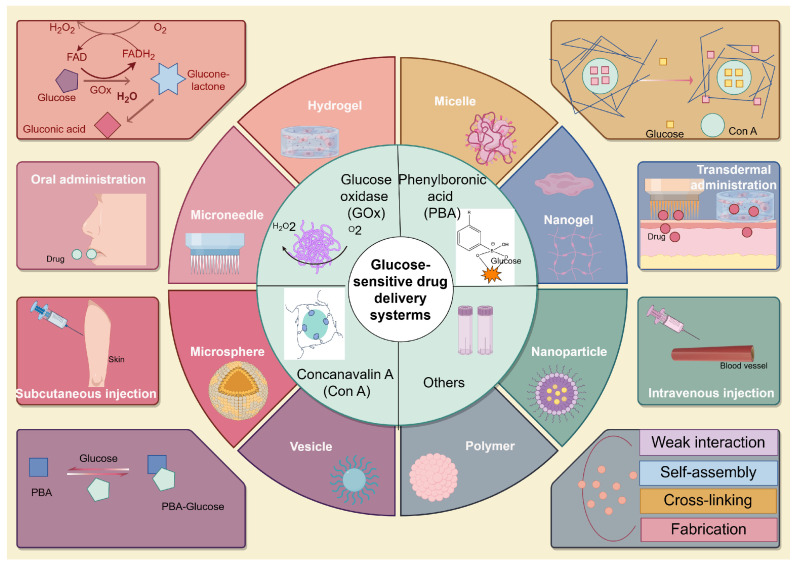
The classification and application of glucose-responsive drug delivery systems. Image created by Figdraw 2.0 (https://www.figdraw.com/, 21 August 2024).

Glucose-responsive drug delivery systems use particular identification components that are sensitive to glucose position changes and are designed to release medication accordingly. The system mainly contains many types of sensitive components: ① the glucose oxidase (GOx) enzyme, which catalyzes the oxidation of glucose; ② phenylboronic acid (PBA), which has the distinct possession of forming reversible covalent compounds with diols (including glucose); ③ the Concanavalin A (Con A), a lectin protein that joins glucose and other sugars; and ④ other sensitive components, such as cells. They are incorporated into drug delivery systems as an identifying element for glucose sensitivity (Figure 2). The environmental sensitivity of these identifying elements contributes to their capability to control drug release in response to glucose concentration. This approach tries to allow targeted drug delivery, in which medication is released according to physiological flare, such as glucose attention. It can be used for minimizing side effects and improving treatment effectiveness for diabetes.

#### 3.3.1. GOx-Based Glucose-Responsive Drug Delivery System

**Figure 3:**
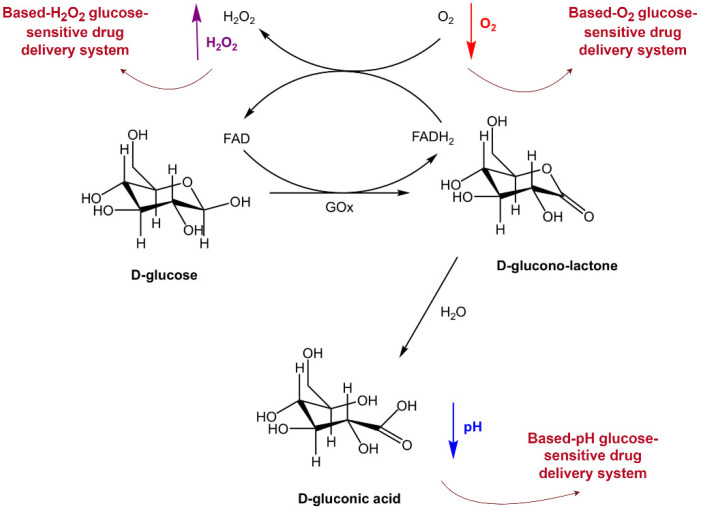
Graphic example of the glucose oxidase-catalyzed oxidation of glucose. Image created with KingDraw (KingDraw Windows, v3.0) and Figdraw 2.0 (https://www.figdraw.com/, 21 August 2024). The colors in the figure are for differentiation only.

GOx is a glucose response system that is widely used. It is an oxidoreductase that particularly catalyzes β-D-glucose. The drug delivery system based on GOx is a homodimer that uses the Enzo subunits with a relative molecular lot of 80 kDa, each compulsory a flavin adenine dinucleotide coenzyme [21]. When combined with a pH-responsive polymer material, GOx catalyzes glucose to produce gluconic acid in a hyperglycemic environment. It can change the pH of the microenvironment. It can bring about a conformational or constructional alteration in the carrier, which facilitates the release of the preloaded medication. Concurrently, the consumption of O_2_ causes the GOx to form an in-situ hypoxic microenvironment, which causes a reduction reaction of some components of the carrier. It can ruin the medication-filling system to release the preloaded drug [22]. In the GOx-based glucose-sensitive drug delivery system, the release of insulin or other hypoglycemic drugs is affected via the glucose in the blood. GOx uses O_2_ as an electron acceptor to catalyze glucose and swiftly consumes O_2_ to cause gluconic acid and hydrogen peroxide (H_2_O_2_). The pH of the microenvironment and O_2_ thinking drops, and H_2_O_2_ attention rises after the oxidation of glucose (Figure 3) [23]. These changes in pH, H_2_O_2_ concentration, and oxygen levels could act as triggers to initiate drug release from the delivery system. The glucose-sensitive dynamic action of glucose oxidase causes a response in the system. Therefore, GOx was introduced into carriers with pH response, O_2_ response, and H_2_O_2_ response to prepare the glucose-responsive drug delivery systems. Table 2 summarizes the drug delivery systems based on GOx developed over the past decade, including pH, H_2_O_2_, and O_2_-sensitive systems. It might be related to the principle of glucose oxidase. Currently, multi-system combined technology has been applied. It could learn from strengths and weaknesses to give full play to the value of GOx and enhance the application to the detection and treatment of diabetes [24].

A notable characteristic of a smart drug delivery system is its responsiveness to the glucose concentration in the surrounding environment. It is crucial for the management of diabetes. GOx plays a pivotal role in facilitating this glucose-responsive mechanism. A closed-loop drug delivery system has been developed that encapsulates GOx, gold nanoparticles (AuNPs), and metformin within a Zeolitic Imidazole Framework-8 (ZIF-8). This reaction leads to a decrease in the surrounding pH, causing the degradation of ZIF-8 and the subsequent release of the encapsulated drug [25].

Glucose-responsive insulin delivery systems have demonstrated efficacy in the treatment of diabetes, especially the insulin delivery system. A notable example was the use of acetate dextran nanoparticles embedded within a porous alginate microgel, which exhibited a rapid response to fluctuations in glucose concentration. This gel has been shown to prolong the release of glucose-reactive insulin and maintain blood sugar control in diabetic murine models for 22 days [26]. Furthermore, mannose ligand-conjugated nanoparticles (NPs) were synthesized using a contraction gel method. They involved alginate, insulin, and glucose oxidase. These NPs released their drug payload exclusively in high glucose concentration solutions while inhibiting drug release in normal and low glucose concentration environments. These findings indicated that the development of glucose-reactive NPs might represent a promising strategy for oral insulin delivery [27]. Additionally, microneedles (MNs) fabricated from poly-L-lysine-modified cationic silk fibroin (SF) exhibited glucose reactivity upon incorporating glucose oxidase. In vivo studies have shown that normal SD rats using this MN had lower insulin release than diabetic rats. Following administration, the blood glucose levels of diabetic rats in the injection group surged to 33.1 mmol/L before gradually decreasing. In contrast, the levels in the patch group initially rose to 21.7 mmol/L at 6 h and subsequently fell to 15.3 mmol/L. This suggested that insulin release from microneedles was triggered by increases in blood glucose concentration [28]. To replicate the activity of natural insulin, materials have been developed to encapsulate insulin and glucose oxidase for delivery. However, a significant challenge remains in achieving the desired rapid and sustained release dynamics. The insulin release of the polymer nanoparticles could be adjusted by modifying the acid-degraded glucan acetate polymers to different degrees. Nanoparticles synthesized from dextran with a high acyclic acetal content (94% of residues) exhibited rapid release kinetics, while those synthesized from dextran with a lower acyclic acetal content (71% of residues) demonstrated a slower release rate. Consequently, the co-formulation of these two materials facilitates both rapid and prolonged glucose-responsive insulin delivery [29].

Furthermore, numerous insulin delivery systems utilizing glucose oxidase have been patented. A novel pH-sensitive polypeptide has been developed that self-assembles into a hydrogel. This system facilitated a glucose-responsive insulin release mechanism, which was employed in the management of diabetes. The polypeptide material could be loaded with glucose oxidase, catalase, and insulin to create a glucose-responsive insulin delivery system [30]. Additionally, ZIF-8 has been combined with glucose oxidase to establish a glucose-responsive system that was subject to patent protection. The findings indicated that the nano complex was prepared with a combination of CIP and GOx encapsulated within ZIF-8 as a nanocore. The nano complex could function as a glucose-responsive nanocomposite microneedle patch. This patch was designed for the treatment of diabetic wounds and to enhance the wound-healing process [31].

GOx possesses a high specific surface area and favorable conductivity. Furthermore, GOx has enzyme-like dimensions and distinctive properties that facilitate functionalization. However, the increased specific surface area of GOx might also contribute to heightened pollution levels. It is imperative to further investigate the stability of GOx, as well as the characteristics of analogous enzymes in vivo. Degradable biocompatible materials represent a significant area of research and are anticipated to be a focal point of future studies [32]. Although base GOx glucose-responsive insulin delivery vectors have been validated in animal models, their clinical application remained elusive [33]. There is a notable deficiency in clinical trial data necessary to assess the toxicity and efficacy of GOx as a glucose-sensitive drug delivery system.

**Table 2:** Application of GOx glucose-sensitive drug delivery system in recent ten years.

Name	Type	Platform	Mechanisms	Animal Model	Reference
Zeolitic imidazole framework-8 (ZIF-8) @insulin&GOx	Based-pH	Nanoparticles	GOx oxidizes glucose to gluconic acid, ZIF-8 releases insulin in an acidic environment	STZ-induced diabetic rats (C57BL/6 mice)	[34]
Insulin/Ca_3_PO_4_-GOx/Cu_3_(PO_4_)_2_ hyaluronic acid microneedle	Based-pH	Microneedle	GOx oxidizes glucose to gluconic acid, the carrier releases insulin when pH decreases	STZ-induced diabetic rats (SD rats)	[35]
GOx assembled with mixed multiple layer-by-layer-insulin microspheres	Based-pH	Microspheres	GOx oxidizes glucose to gluconic acid, hydrolyzes amine, convert anionic polymer to cationic polymer, release insulin	STZ-induced diabetic rats	[36]
Polylysine-modified cationized silk fibroin—insulin delivery Microneedles	Based-pH	Microneedles	GOx makes the system glucose reactive, oxidizes glucose to gluconic acid, pH decreases, insulin release increases	STZ-induced diabetic rats	[28]
Arginine@ Zinc metal-organic framework-GOx gel	Based-pH	Hydrogel	GOx oxidizes glucose to gluconic acid, pH decreases	STZ-induced diabetic rats	[37]
Self-delivery glucose oxidase @ manganese sulfide nanoparticles	Based-pH	Nanoparticles	GOx catalyzes glucose, pH decreases, manganese ions (Mn^2+^) and hydrogen sulfide (H_2_S) release	STZ-induced diabetic rats	[38]
Glucose-responsive multifunctional metal-organic drug-loaded hydrogel	Based-pH	Hydrogel	GOx catalyzes glucose, pH decreases, zinc ions release	High-fat diet and STZ-induced diabetic rats (BALB/c mice)	[39]
Sodium bicarbonate and GOx-insulin microneedles	Based-pH	Microneedles	GOx catalyzes glucose, pH decreases; The protons react with NaHCO_3_ to form CO_2_, create pressure within the pores, rupture the membrane, release insulin	STZ-induced diabetic rats (SD rats)	[40]
Insulin@ PEGylated bilirubin/GOx nanoparticles	Based-H_2_O_2_	Microneedles	GOx catalyzes glucose, pH decreases, releases insulin	STZ-induced diabetic rats (C57BL/6 db/db mice and BALB/c mice)	[41]
L-arginine (L-Arg)-coupled chitosan and GOx-modified hyaluronic acid hydrogel	Based-H_2_O_2_	Hydrogel	Glucose and L-arginine mediate the sustained release of H_2_O_2_ and NO in hyperglycemic environments	STZ-induced diabetic rats (BALB/c male mice)	[42]
POD-like Fe_2_(MoO_4_)_3_/ GOx polymers	Based-H_2_O_2_	Polymers	GOx oxidizes glucose to gluconic acid and H_2_O_2_; H_2_O_2_ converts into highly oxidative center dot OH via catalysis of POD-like Fe_2_(MoO_4_)_3_	STZ-induced diabetic rats	[43]
Chitosan (CS)/MnO_2_-GOx nanoparticles	Based-H_2_O_2_	Nanoparticles	CS as a catalyst; GOx oxidizes glucose to gluconic acid and H_2_O_2_; MnO_2_ consumes glutathione to produce Mn^2+^; Mn^2+^ mediates Fenton-like reactivity	STZ-induced diabetic rats (C57BL/6 db/db mice)	[44]
Copper ion guanosine monophosphate/GOx bifunctional Hybrid Nanoflowers	Based-H_2_O_2_	Nanoflower	GOx oxidizes glucose to gluconic acid and H_2_O_2_	NO animal modle	[45]
Berberine and epigallocatechin gallate coated with manganese dioxide nanoshells (MnO_2_) and GOx	Based-O_2_	Polymers	GOx oxidizes glucose to gluconic acid and H_2_O_2;_ MnO_2_ decomposes into O_2_	STZ-induced diabetic rats (BALB/c mice)	[46]
GOx and catalase nano enzyme-chitosan hydrogel complex	Based-O_2_	Hydrogel	GOx oxidizes glucose to gluconic acid and H_2_O_2_	STZ-induced diabetic rats (SD rats)	[47]
Calcium phosphate -GOxCu_2_O/Pt nanoreactor	Based-O_2_	Polymers	GOx oxidizes glucose to gluconic acid and H_2_O_2,_ H_2_O_2_ converts to highly active hydroxyl radicals through the triple enzyme-like activity of Cu_2_O/Pt, the acidic microenvironment generated by gluconic acid	STZ-induced diabetic rats (SD rats)	[48]
GOx and insulin glucose-responsive vesicles	Based-O_2_	Microneedle	Local hypoxia caused by glucose-enzyme oxidation, vesicles dissociate, release insulin	STZ-induced diabetic rats (C57BL mice)	[49]
Poly[4-(4,4,5,5-tetramethyl-1,3,2-dioxaborolan-2-yl) benzyl acrylate]-b-poly[2-(dimethylamino)ethyl methacrylate] polymer	Based-multi sensitive	Microneedle	GOx oxidizes glucose to gluconic acid and H_2_O_2,_ stimulates the drug release	STZ-induced diabetic rats (SD rats	[50]
GOx/mesoporous polydopamine (MPDA)/Fe-doped carbon dots (Fe@CDs) nanoreactor	Based-multi sensitive	Nanoreactor	Cascade nanozyme GOX/MPDA/Fe@CDs self-supply H_2_O_2_ and H^+^, break H_2_O_2_ and pH limits	STZ-induced diabetic rats (BALB/c mice)	[51]
Self-tandem bio-heterojunctions (bio-HJs) consisting of molybdenum disulfide (MoS_2_), graphene oxide (GO), and GOx are constructed on orthopedic polyetheretherketone implants	Based-multi sensitive	Polymers	GOx excreted glucose to produce H_2_O_2_, bio-HJs produced hyperthermia under near-infrared light	STZ-induced diabetic rats (BALB/c mice)	[52]
Ciprofloxacin hydrochloride (CIP)/GOx @zeolite imidazole framework-8 (ZIF-8) microneedles	Based-multi sensitive	Microneedles	GOx mediates glucose consumption and H_2_O_2_ production in combination with Zn^2+^ and CIP	STZ-induced diabetic rats (SD rats)	[53]
Hypoxia and H_2_O_2_ dual-sensitive diblock copolymer GOx-insulin delivery device	Based-multi sensitive	Microneedles	GOx oxidizes glucose to gluconic acid and H_2_O_2_, releases insulin.	STZ-induced diabetic rats (C57BL/6 db/db mice)	[54]

#### 3.3.2. PBA-Based Glucose-Responsive Drug Delivery System

**Figure 4:**
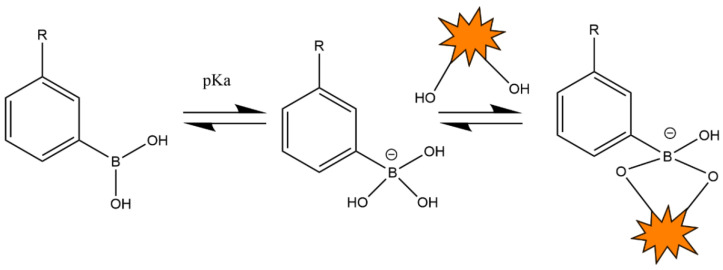
Schematic diagram of the combination of PBA structure and adjacent diol structures.

PBA is a synthetic molecule that is hydrolyzed in an aqueous solution to form electrically neutral and electronegative forms. Among these, the electroneutral PBA has poor hydrophilicity, while electronegative PBA has better hydrophilicity. PBA, mainly in its electronegative form, has a special binding effect on cis-1,2-/1,3-diols. Boric acid and o-glycol-containing substances can rapidly esterify in an aqueous environment to form borate bonds (Figure 4). PBA exists in the dynamic form of an uncharged triangular planar state and tetrahedral form with an anion. The o-diol structure of glucose allows PBA to dynamically amalgamate with the anionic tetrahedral structure. Hence, the stability is changed to the anionic PBA type, improving the hydrophilicity and raising the negative charge tightness. When glucose concentrations decrease, this equilibrium shifts to the left. Furthermore, when multiple compounds of o-glycols are present, boric acids with tetrahedral structures preferentially bind to compounds with higher bonding constants. The charge and hydrophilic transitions are induced by the equilibrium shift of phenylboronic acid in conjunction with glucose, along with the competitive interactions between glucose and other glycols. It can facilitate the development of numerous glucose-sensitive systems based on phenyl borate. These systems are increasingly utilized in the formulation of glucose-sensitive drug delivery carriers.

PBA is a weak Lewis acid with a pKa range of 7.8 to 8.6. The pKa value of unmodified phenylboronic acid usually exceeds 8, resulting in the majority of PBA being in a unionized state under physiological conditions (pH = 7.4). The binding of unionized PBA to glucose is notably weak, leading to a very low glucose response of PBA under physiological conditions. In response to this limitation, the researchers made several attempts. The launch of strong electron-removed groups on PBA (introduction of nitro or halogen groups at the meta-site of boric acid, para-introduction of carboxyl groups) and the introduction of nitrogen atoms around phenylboronic acid to form B-N coordination or combination of PBA with diols, these methods can form phenyl borate could reduce the pKa value of PBA and improve its binding capacity to glucose [21].

Currently, phenylboronic acid derivatives, including 3-aminomethyl phenylboronic acid and 3-Fluoro-4-carboxy-phenylboronic acid, are extensively used. They are mainly applied in the hydrogel response system in response to glucose, among which injectable hydrogels and external hydrogels have been the research hotspots in recent years. Therefore, the review sums up the application of PBA in glucose-sensitive hydrogel systems in the past ten years (Table 3).

The rapid response and controlled drug release insulin delivery system is an essential diabetes therapeutic platform. Microneedles (MNs) with PBA-modified methacrylate hyaluronic acid as the delivery carrier. These MNs consisted of a dynamic bond between PBA and gluconic acid-modified insulin (Gins) as a controlled release component. The efficacy of the insulin MN patch was validated in a rat model of STZ-induced T1D. This study offered insights into simplifying the concept of controlled-release insulin MN patches [55]. Additionally, a glucose-sensitive MN was developed utilizing PBA-modified chitosan particles. The MN was in conjunction with a polyvinyl alcohol/polyvinylpyrrolidone hydrogel. This MN facilitated the efficient delivery of insulin, thereby enabling more sustained blood glucose control [56]. Furthermore, a double-cross-linked insulin micelle was identified. The micelle was based on a triblock polymer of O-ethylene glycol and PBA. This micelle was capable of continuously releasing insulin [57].

In addition, the glucose-responsive drug delivery system developed using PBA has been patented. A glucose-responsive double-layer crosslinked polymer micelle drug delivery system has been successfully prepared. This switchable glucose-reactive double-layer crosslinked polymer micelle drug delivery system. It could enhance stability and mitigate the risk of sudden release. The glucose-responsive double-layer could minimize glucose competition reactions, reduce the likelihood of hypoglycemia, and facilitate long-term cycling [58]. Furthermore, a glucose-responsive gel was composed of n-isopropyl methyl acrylamide, n-isopropyl acrylamide, N,N-dimethyl acrylamide, and N,N-dimethyl acrylamide. The gel was paired with a PBA monomer and a hydroxyl monomer, which had potential applications in insulin drug delivery devices [59].

At present, subcutaneous insulin injections represent the sole therapeutic option for T1D and T2DM patients. However, the administration of insulin via injection is often associated with considerable discomfort for patients. In response to this issue, nasal mucosal administration has emerged as a novel therapeutic approach. This method offered protection for insulin against extensive first-pass metabolism and enzymatic degradation within the gastrointestinal tract. To enhance the permeability of the therapeutic agent, a PBA-functionalized dextran nano platform was constructed. It is formed using a dynamic borate formed from PBA and diol. Compared to unformulated insulin, these nanoplatforms exhibited superior loading capacity and significantly enhanced endocytosis [60].

PBA exhibits numerous advantageous characteristics, including system stability and long-term preservation. However, despite the in vitro and in vivo research demonstrating the favorable biocompatibility of PBA, the assessment of PBA-based glucose-sensitive drug delivery systems remains predominantly confined to preclinical studies. The main research goal of PBA is to develop non-toxic side effects in the treatment of diabetes, thus allowing PBA to be effectively cleared from the body. It ensures that PBA becomes the best biocompatible and biodegradable drug carrier [61].

**Table 3:** Summary of research on PBA in glucose-responsive hydrogels in the past ten years.

Type of PBA	Materials	Function	Type of Hydrogel	Reference
3-aminomethyl phenylboronic acid	Hyaluronic acid; polyethylene glycol diacrylates; myricetin	Hydrogel has exhibited great potential for diabetic wound treatment	Hydrogel dressings	[62]
3-aminomethyl phenylboronic acid	Hyaluronic acid methacrylate; phenylboronic acid; hyaluronic acid derivative; catechin	Hydrogel with potential for application in diabetic wound treatment	Hydrogel dressings	[63]
Phenylboronic acid	Folliculin-interacting protein 1; hyaluronic acid; phenylboronic acid; fulvic acid	Promising strategy hydrogel for hydrogel chronic diabetic wound repair	Hydrogel dressings	[64]
4-(Bromomethyl)-Phenylboronic acid	Gallic acid; chitosan; poly (ethylene glycol) diacrylate; polyethyleneimine; phenylboronic acid	Hydrogel with glucose-responsive hyperglycemia regulation and antioxidant activity for enhanced diabetic wound repair	Hydrogel dressings	[6]
Formylphenylboronic acid	Polyvinyl alcohol; nature-abundant proteins (bovine serum albumin, egg albumin, casein); formylphenylboronic acid	Injectable and potential as smart insulin for in vivo applications shortly	Injectable hydrogel	[65]
3-Fluoro-4-carboxy-phenylboronic acid	3-fluoro-4-carboxy-phenylboronic acid-grafted polylysine; natural guar gum	Maintaining performance via glucose-responsive transdermal insulin delivery	Hydrogel dressings	[66]
3-Fluoro-4-carboxy-phenylboronic acid	Galactosyl; 3-fluoro-4-carboxy phenylboronic acid	Hydrogels that increase the crosslinking density can slow the spread of insulin in the body and control the release of insulin	Injectable hydrogel	[67]
4-(2-Acrylamidoethylcarbamoyl)-3-fluoro-phenylboronic acid	Biocompatible silk fibroin (sf); 4-(2-acrylamidoethylcarbamoyl)-3-fluorophenylboronic acid; acrylamide	Regulates the epidermal layer and releases insulin autonomously, corresponding to the glucose change pattern	Hydrogel dressings	[68]
3-(Acrylamido)phenylboronic acid	N-isopropylacrylamide; 3-(Acrylamido) phenylboronic acid; Alginate	In response to changes in glucose concentration, reversible sol-gel conversion is generated to achieve self-regulated release of insulin	Hydrogel dressings	[69]
3-(Acrylamido)phenylboronic acid	N-isopropylacrylamide 99% stabilized; 2-(Dimethylamino) ethyl methacrylate; 3-(Acrylamido)phenylboronic acid	pH-dependent insulin release patterns	Hydrogel	[70]
4-Carboxy-3-fluorophenylboronic acid	2,5-Dimethylbenzoic acid; N-(2-Hydroxyethyl) maleimide; 3-Pyridylboronic acid, 2-(Bromomethyl)benzoic acid, 3-(Bromomethyl)benzoic acid, Hydroxybenzotriazole monohydrate; PEG	Improve responsiveness translates to more rapid blood glucose correction in a rodent diabetes model	Injectable hydrogel	[71]
4-Carboxy-3-fluorophenylboronic acid pinacol ester	4-Armpolyethylene glycol; 4-Carboxy-3-fluorophenylboronic acid pinacol ester	Accelerate the release of insulin to glucose	Injectable hydrogel	[72]
4-Carboxyphenylboric acid	Quaternary ammonium chitosan; Dihydrocaffeic acid; l-arginine; oxidized hyaluronic acid-dopamine; methacrylated poly (vinyl alcohol) (methacrylated PVA); phenylboronic acid; gallium porphyrin; 3-Amino-1,2 propanediol; Insulin	Excellent biocompatibility, slow drug release	Hydrogel	[73]
Carboxy phenylboronic acid	Natural silk fibroin protein; carboxy phenylboronic acid	Modification of silk fibroin into a glucose-responsive hydrogel platform for regulated and functional insulin delivery application	Injectable hydrogel	[74]
4-Vinyl-phenylboronic acid	Intelligent cellulose; 4-Vinyl-phenylboronic acid	The complex hydrogel self-regulates insulin release under different concentrations of glucose	Hydrogel	[75]
3-Acrylamidophenylboronic acid	Acrylamide or N-isopropylacrylamide; N, N′-methylenebis; 3-Acrylamidophenylboronic acid; N-(3-dimethylaminopropyl)	Method for determining the amount of bound glucose in hydrogels	Hydrogel	[76]

#### 3.3.3. Con A-Based Glucose-Sensitive Drug Delivery System

**Figure 5:**
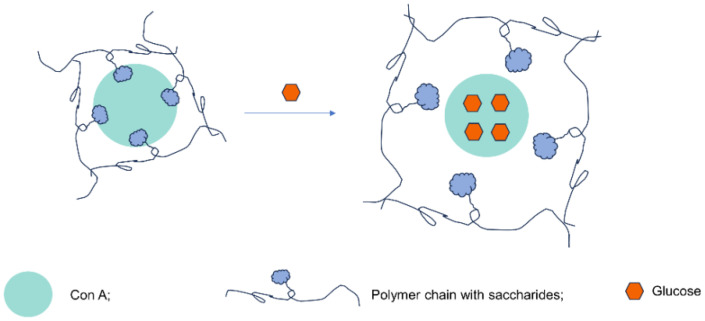
Schematic diagram of Con A in response to glucose.

The third category of glucose-sensitive compounds is glucose-binding proteins [21]. Concanavalin, also known as Concanavalin A (Con A), is a plant lectin extracted from *Phaseolus vulgaris* L. (lectin refers to a class of proteins with a very diverse structure, which is characterized by the ability to specifically bind carbohydrates). Con A is a tetramer globulin, which has a high affinity for sugars rich in mannose [77]. The sugar-binding site of Con A can bind hydroxyl groups α-D-mannopyranose, α-D-glucose pyranose, and other non-reducing sugars at C-3, C-4 and C-6. Therefore, Con A could also be used in glucose-responsive intelligent systems (Figure 5). Con A is usually fixed in the polymer matrix by covalent bonds to the glycosylated portion of the polymer chain. This obsession causes a three-solid arrangement where Con A is tied to the medication to be brought. When a polymer matrix containing Con A and the medication is unconcealed to a glucose response, complimentary glucose particles are current in the solution. When glucose positions are exalted (show hyperglycemia), the complimentary glucose in the response contends with the glycosylated part of the polymer to unite to Con A. The glucose molecule preferentially binds to Con A rather than the sugar bound to the polymer. Since Con A separates from the polymer matrix under the deed of glucose, it brings about the release of medication co-fixed with Con A. This is an example of how biometric elements like Con A can be integrated into smart materials for targeted drug delivery applications.

There are two primary methods for self-examining lifeblood glucose in diabetic unwell persons: blood glucose monitoring (BGM) and continuous glucose monitoring (CGM). CGM could detect the patient’s blood glucose level in real-time and find hidden hyperglycemia and hypoglycemia. That might not be easy to detect by traditional detection methods. It has become a new trend in lifeblood glucose checks. A silver-doped hydrogel sensor is proposed in the field of continuous glucose detection. It is prepared by combining Con A-based glucose-sensitive hydrogel with green synthetic silver particles on a laser direct-writing graphene electrode. Capable of measuring glucose levels in a reproducible and reversible manner over a glucose concentration range of 0 to 30 mmol/L. The sensor is the best enzyme-free glucose sensor available [78]. Additionally, a capacitive glucose sensor exhibiting high linearity and a broad detection range has been introduced. The sensor is based on a glucose-sensitive Con A hydrogel and a serpentine coplanar electrode constructed from low melting point metal. The sensor hydrogel could accomplish high linearity (R^2^ = 0.94) with a susceptibility over a glucose attention series of 0 to 20 mmol/L and excellent linearity (R^2^ = 0.84) even at a glucose concentration range of 0 to 30 mmol/L. The excellent reversibility and long-word firmness produced by Con A have a fantastic possibility for a lifeblood glucose check [79].

Conversely, a hydrogel composed of Con A and glucose units is achieved through specific binding and double cross-linking of amide bonds. It has been developed to extend the half-life of the anti-diabetic peptide medication exenatide and to sustain blood glucose homeostasis. The nano gel could protect the peptide drug from degradation in plasma, improving exenatide’s therapeutic effect [80]. However, it has been reported that Con A poses a significant risk of leakage from the hydrogel matrix. To improve this limitation, a thermally responsive co-formation matrix has been established between Pluronic F-127 and structurally robust chitosan. The matrix was coupled by 1-(3-Dimethylaminopropyl)-3-ethylcarbodiimide (EDC) and N-hydroxysuccinimide (NHS). The structural stability and integrity of the hydrogel have been fully verified, but further exploration is needed for in vivo animal models [81]. Phthalocyanine is a blue macrocyclic compound with an intermolecular pi-pi stacking structure. A glucose-sensitive lipid system was prepared to modify the surface with 1-(3-Dimethylaminopropyl)-3-ethylcarbodiimide hydrochloride (EDAC): NHS (1:1) to conjugate Con A. After the conjugation of Con A with cochleate, in vivo studies improved blood glucose levels.

This system significantly improved diabetic rats by protecting pancreatic β cells [82]. Insulin is the important medication in T1D clinical care. However, the oral bioavailability of insulin is impoverished because of insulin’s incapability to oppose gastrointestinal ingestion. An oral drug delivery system for diabetes has been developed. Con A in konjac glucomannan nanoparticles was prepared by cross-linking, which loaded with insulin as an oral insulin delivery system. In vivo trials have shown its safety and biocompatibility [83]. Diabetic complications are endocrine diseases characterized by inflammation, and it is critical to minimize the amount of inflammatory markers in patients with diabetic complications while maintaining average blood glucose levels. In this study, a responsive nanosystem with anti-inflammatory effects was developed for smart oral insulin [84]. Furthermore, reversible crosslinked nanoparticles composed of Con A and glucomannan have been patented. This innovative nanoparticle has an effective glucose regulation function. It could be used as a low-toxicity transport system with specific long-term hypoglycemic effects. Additionally, these nanoparticles could enhance the blood glucose control efficacy of the insulin oral transport system [85]. It recommends that Con A is possible not only in glucose diagnosis but also in diabetes treatment.

At present, most of the research on glucose-sensitive systems with Con A is insulin-loaded. However, there are a series of problems in the practical application of the Con A system, including leakage, stability and solubility in water, length of response time, biological toxicity, and biological activity. Studies have shown that Con A may cause acute damage to the kidneys, livers, and other organs of animals. These are urgent problems to be solved in the Con A-based glucose-responsive insulin delivery system. To avoid leakage, Con A is covalently bound to the polymer. A specific requirement for hydrogel synthesis has been reported to be to keep the pH below 9 during the reaction. It can reduce the inactivation of Con A during conjugation. Furthermore, when searching for and constructing delivery materials with precise structures, providing comprehensive information on the most promising systems could provide better biocompatibility and bioactivity, including biocompatibility and biostability/biodegradability under physiological conditions [77].

#### 3.3.4. Others-Based Glucose-Sensitive Drug Delivery System

In addition to the GOx, PBA, and Con A conventional glucose response factors, additional glucose-reactive systems have been investigated. An insulin-delivery microneedle (MN) array patch has been reported to possess glucose-reactive properties, which were loaded with red blood cell vesicles. The MN incorporated glucose transporters (GLUTs) that facilitated the modification of insulin to form Glu-Insulin. In hyperglycemic conditions, elevated glucose concentrations in the interstitial fluid could displace glucose insulin through competitive interactions with GLUTs. It resulted in the rapid release of glucose–insulin and subsequent regulation of blood glucose levels. This MN patch offered a simplified approach to glucose management and was particularly suitable for patients with T1D and T2DM [86]. Self-regulation and insulin delivery that mimics the natural function of the pancreas is a long-term goal of diabetes treatment. Two primary strategies to achieve this include glucose-responsive insulin delivery and islet cell transplantation therapy. A biodegradable, partially oxidized alginate carrier designed for glucose-responsive nanoparticle delivery has been reported. This injectable system was characterized by a 0.5 mm diameter and 2.5% oxygenated microgel. It was capable of providing continuous blood glucose control for 10 days. These findings underscored the potential of biodegradable vectors in the management of drug and cell delivery for diabetes treatment [87].

Chemical reactions represent a multifaceted approach to the development of biopharmaceuticals. The synthesis of lectin mimics serves as a complementary advancement to the natural lectin Con A. The synthetic pathway for s-Lectin has been optimized. A novel glucose-responsive microgel was achieved by encapsulating s-Lectin within a poly (N-isopropyl acrylamide) crosslinking network. The resulting s-Lectin microgel exhibited swelling behavior in response to glucose concentrations ranging from 0 to 30 mmol/L. Thereby, it has the specific recognition of glucose. This gel holds promise for monitoring blood glucose levels [88]. Fluorescent-based sensors are particularly promising for continuous in vivo glucose monitoring (CGM), which facilitates wireless transdermal transmission and prolonged functionality within biological systems. However, current fluorescent-based sensors face challenges in maintaining their position at the implant site for extended durations and effectively responding to fluctuations in blood glucose concentrations. The study revealed polyethylene glycol (PEG)-bound polyacrylamide (PAM) hydrogel fibers. Furthermore, a glucose-cleavable linker was designed based on the structures of hydrazone and thiazolidine. It had reactivity to glucose in vitro. Aliphatic hydrazone and thiazolidine moieties were conjugated to the LysB29 side chain of insulin through pH-controlled acylation, rendering GRI responsive to thiazolidine in vitro. This research might offer novel insights into biopharmaceuticals in the domain of glucose-responsive drug delivery [89].

These studies remain in the exploratory phase. They currently lack comprehensive investigation in comparison to the GOx, PBA, and Con A systems. Nevertheless, these concepts have the potential to enhance the glucose response system, thereby improving its application in the monitoring and treatment of diabetes.

### 3.4. Application of Glucose-Response Drug Delivery System

Recent research on medication delivery systems, particularly concerning insulin, has led to the development of numerous glucose-responsive materials and technologies. These include hydrogels, nanoparticles, microneedles, and other systems. This review summarized the advantages and disadvantages of glucose-responsive drug delivery systems (Table 4). According to the mode of administration, the glucose response drug delivery system could be divided into transdermal administration, injection administration, and oral administration.

#### 3.4.1. Based on Transdermal Administration Glucose-Sensitive Drug Delivery System

##### Hydrogels

Non-restoring injuries originating from diabetes are one of the serious problems of diabetic persons. It is the chief kind of persistent unmanageable wound in clinical. In recent years, glucose-responsive dressings prepared from the hyperglycemic microenvironment of diabetic wounds have shown great potential. Glucose-responsive hydrogel injury dressings have a wide series of requests in diabetic wounds. Glucose-sensitive hydrogel wound dressings can play a role in the wound microenvironment of hyperglycemia. While pH-responsive hydrogel wound dressings can effectively sense pH changes in the wound microenvironment. Furthermore, dual-sensitive hydrogel injury dressings could apply synergistic belongings in response to the complex microenvironment of wounds. It could help with injury restoration. Therefore, responsive dressings based on the wound microenvironment could release drugs at different times and places through different signals. It can provide research arrangements for exact remedies to a certain extent.

Hydrogels are a form of highly hydrophilic three-solid network arrangement that comes together. Hydrogels increase quickly in liquid and can continue in a large amount of liquid without melting in this enlargement state. As a new type of medical dressing, the application range of hydrogels is expanding annually [100]. There exists a diverse array of hydrogel formulations, and numerous new hydrogel materials are continuously being developed. Their purpose is becoming more faultless [101].

The active ingredients from natural products have been shown to have a wide range of potential in diabetes. Among them, polysaccharides are used for treatment and as cross-linking agents in hydrogels. The polysaccharide extracted from *Bletilla striata* was cross-linked with bioactive natural polymers carbomer 940 and carboxymethyl chitosan to prepare a polysaccharide hydrogel. This hydrogel has a porous structure and good mechanical and moisturizing properties. Furthermore, the STZ-induced mouse diabetes model verified that this hydrogel could significantly promote wound healing [102]. Other busy components of TCM are also used in hydrogels. In this study, gelatin methacryloyl (GelMA) was modified with 4-carboxyphenylboronic acid (CPBA) to synthesize GelMA derivatives (GelMA-CPBA) and then photo crosslinked with (-)-epigallocatechin-3-gallate (EGCG) to form GelMA-CPBA/EGCG (GMPE) hydrogels. As glucose levels rise, more EGCG is released due to the dissociation of boron ester bonds. The hydrogel could improve collagen deposition and tissue remodeling during diabetic wound healing [103]. Myricetin (MY) was loaded into glucose-sensitive PBA modified into hyaluronic acid (HA) and polyethylene glycol diacrylate (PEG-DA) hydrogel matrix through the polyphenol group of MY, which realized the release of MY, effectively remodeled the unfavorable diabetes wound microenvironment. This novel glucose-sensitive antioxidant crossbreed hydrogel has a great possibility for diabetic injury [62]. A glucose-sensitive hydrogel has been patented. The hydrogel contained N-(2-D-glucose) acrylamide, acrylamide, (N, N′-methylene bisacrylamide, and Con A. The hydrogel was prepared by polymerization under the action of a redox initiator in a thioglycolic acid aqueous solution. This hydrogel provided an increased release of insulin during the initial stage of glucose emergence [104].

However, there remains a notable gap between hydrogel dressings and conventional wound dressings. Over the twelve months, researchers at dwellings and overseas have dedicated time and strength to solving this difficulty. Primarily, exact sensitive capability and good biocompatibility are still the chief goals of future sensitive dressing. Most flow use of polymer hydrogels and new wound dressings require further advancement, such as peptide hydrogel dressings. Additionally, it is important to note the pH of chronic wounds tends to be alkaline. Most pH-responsive hydrogel dressings are aimed at acidic environments. Consequently, there is a need to investigate hydrogel dressings capable of delivering drugs on demand in the alkaline conditions characteristic of chronic wounds. Finally, the procedure of injury restoration is complex and different. Injury dressings can be programmed and treated according to the relevant characteristics of each wound. They are more in line with the requirements of personalized medicine. However, hydrogel materials are inherently weak, including low strength and poor toughness. This has become a bottleneck problem limiting their application.

##### Microneedles

The emergence of microneedling represents a significant advancement in transdermal drug delivery systems. Microneedles can be made of metals, silicon compounds, and polymers. Glucose-responsive microneedling based on PBA, which can painlessly bring insulin and self-control insulin set free based on blood glucose levels. It is thought to be encouraging in the care of diabetes. Based on the modified poly-L-Lysine and polyvinyl alcohol, an insulin-supported phenyl borate cross-linked microneedle patch with polyzwitterionic properties was constructed. By regulating the amount of positively charged amino groups after protonation, this well-designed microneedle patch has improved glucose-responsive insulin delivery performance [105]. The microneedles were composed of HA polymer, dopamine, and 4-amino-3-fluorobenzoborate. Catechol groups in the dopamine unit in microneedles form covalent cross-links through autoxidation. It is dynamically cross-linked with PBA through complexation. Due to the interaction of HA with insulin, the hydrogel polymer also retains the natural structure and biological activity of insulin. This gives it great potential for clinical translation [106]. A gel–AFPBA–insulin hydrogel microneedle dressing was prepared with biocompatible methacrylate gelatin, glucose-responsive monomer 4-(2-acrylamide ethyl carbamoyl)-3-fluorophenyl boronic acid (AFPBA) and glucose–insulin. The hydrogel microneedle dressing has sufficient mechanical properties, high biocompatibility, and glucose-responsive insulin release behavior when exposed to different glucose solutions. Furthermore, the hydrogel microneedle dressing has strong adhesion to the skin compared with the hydrogel without microstructure. Microneedling dressings could increase the restoration procedure of diabetic injury and lessen inflammatory responses. It could improve collagen sworn statement in regenerated matter areas and improve lifeblood glucose control in animals [107]. A material called 3-fluoro-4-carboxyphenylboronic acid (FCPBA) grafted poly-L-lysine (PLL-PBA) was created. This material was combined with natural guar gum (GG) to form PLL-PBA/GG hydrogels. The hydrogel was used to prepare PLL-PBA/GG microneedles using a micro mold casting method. The microneedles were formed by taking advantage of the phenyl borate bond formed between the FCPBA group on PLL-PBA and the diol on GG. Due to the simple and gentle preparation conditions of microneedling, the biological activity of insulin is greatly preserved. The microneedle has a uniform morphology and moderate piercing ability. It has good glucose-responsive insulin release capacity. Additionally, they demonstrate BGL-reducing and maintenance properties through glucose-responsive transcutaneous insulin delivery [66]. These patches offer a potential treatment for diabetic wounds.

Several microneedles have been patented. Microneedles were constructed by adding PBA or its derivatives into methacrylate hyaluronic acid skeleton carrier material. The glucose-sensitive mechanism was predicated on the reversible covalent bond established through the grafting of PBA onto hyaluronic acid methacrylate, as well as the interaction between o-diol and gluconate insulin. This microneedle was characterized by its straightforward preparation process. It could intelligently release insulin [108]. Furthermore, additional research demonstrated that poly-L-lysine modified with PBA could form complexes with insulin possessing a diol structure. These microneedles hold potential for the formulation of pharmaceuticals aimed at diabetes treatment [109]. Empirical studies indicated that the design of microneedles might yield favorable glucose release performance.

##### Others

Microneedles have been extensively developed for application in transdermal insulin delivery systems. However, the synthesis of vesicles is also of significant importance. A microneedle comprises a glucose- and pH-responsive supramolecular polymer vesicle (PV) along with a water-soluble polymer functioning as a substrate. This PV is formed through a host–guest inclusion complex involving pH-responsive water-soluble aromatics (WP5) and glucose-sensitive PBA. It could load insulin and GOx. Drug-loaded PVs exhibited a rapid response to elevated glucose levels. It was characterized by the dissociation of the PV and the swift release of encapsulated insulin. This innovative insulin delivery device was synthesized from multifunctional vesicles. It could hold considerable potential for application in diabetes [110]. In the context of blood glucose measurement, a glucose-reactive fluorescence unit could alter its fluorescence by the glucose concentration in bodily fluids. This unit was designed to adhere to the user’s skin in the form of a patch. Blood sugar levels in bodily fluids were assessed by analyzing the intensity of the detected fluorescence or through fluorescence imaging. This sensor was capable of continuous blood sugar monitoring and had the potential to reduce side effects, such as pain [111].

Although transdermal administration is very convenient and effectively regulates blood sugar levels, the release of insulin remains suboptimal [112]. The glucose-responsive microneedling still faces the challenges of complex insulin-loading processes or insulin denaturation. Additionally, further verification is needed for the in vivo biocompatibility of sensitive systems. Future research should prioritize comprehensive clinical research to address these issues.

#### 3.4.2. Based on Injection Administration Glucose-Sensitive Drug Delivery System

##### Hydrogels

It is well known that insulin is the strongest of all hypoglycemic drugs. Therefore, insulin injections become one of the options for diabetics. However, due to the various deficiencies of insulin, more injectable insulin in response to glucose has been studied based on the better effect of insulin. PBA-dialdehyde sodium alginate was obtained by grafting APBA with sodium alginate, which is abundant in nature. PBA-dialdehyde sodium alginate could form injectable self-healing hydrogels with carboxymethyl chitosan and gallic acid through dynamic imide and borate bonds. The prepared polysaccharide-based hydrogels showed a three-dimensional porous structure. The hydrogels have good injectability, self-healing, water absorption, and mechanical properties [113]. A low molecular weight hydrogel functionalized with PBA was used as a glucose-sensitive insulin release carrier. The gel could absorb solvents in a buffer aqueous solution with a pH of 8–12, while the sodium salts of the gel form hydrogels at a physiological pH of 7.4. A change in gel morphology due to the addition of glucose indicated glucose-reactive swelling of the hydrogel [114].

Chitosan-based injectable hydrogels were designed and prepared for the precise release of insulin. The hydrogels were dynamic cross-linked double reversible covalent bonds (imine and phenyl borate). The hydrogel contains twofold glucose sensors/response components with high sensitivity and rapid responses. It could change the glucose release position collectively. The hydrogel system showed outstanding glycemic control in glucose tolerance tests, maintaining GBLs in the usual series for up to 11 days after one measure [115].

Currently, injectable hydrogels remain in the research phase. Future iterations of injectable hydrogels must exhibit excellent biocompatibility. Furthermore, these hydrogels should be capable of flowing under moderate pressure and gelling rapidly at the target site. Additionally, the mechanical properties of the hydrogels must be promptly enhanced post-injection to ensure sufficient integrity and strength [116].

##### Polymers

Traditional insulin injections do not effectively control the release of insulin, resulting in unstable blood sugar levels in diabetics. A novel on-demand glucose-responsive polymer system was constructed to achieve long-term glucose regulation and reduce the risk of hypoglycemia. It could be self-assembled into nanoparticles by dynamic covalent bonds between two polymers: fluorophenyl boronic acid grafted polymer and polyol polymer. Insulin is loaded during assembly. The nanoparticles show excellent glucose reactivity in vitro, and insulin set free is powerful at different glucose attention. After in vivo treatment in T1D mice, the BGL regulation time was prolonged, and the risk of hypoglycemia was reduced. The gentle preparation of nanoparticles and excellent glucose control provide an optional diabetes treatment for further clinical applications [117]. Furthermore, the polymer exhibited binding affinity to glucose-reactive mesoporous bioactive glass nanoparticles (BGNs), facilitating their application in the transdermal delivery of insulin. Insulin was encapsulated within the BGNs, whose surfaces were pre-modified with a complex enzyme layer (CEL). This CEL comprised polyethylenimine (PEI), GOx, and catalase (CAT). The polymers facilitated self-regulation and enabled painless administration. The synthesized polymer demonstrated an enhanced hypoglycemic effect while concurrently presenting a reduced risk of hypoglycemia in diabetic rat models [118]

A novel material consisting of glucose-sensitive porous microspheres and polymers has been granted a patent. The porous microspheres in the complex were PBA and glycolic acid, which were with an average diameter of 1–20 μmol/L. The polymer was hyaluronic acid containing catechol. This complex was used in insulin implantable products of long-acting sustained-release drug formulations [119].

##### Others

In comparison to random polymers, the synthesis of block polymers facilitates the preparation of core-shell nanoparticles. That exhibits enhanced glucose sensitivity and increased insulin loading capacity. Specifically, an insulin-supported poly (3-acrylamidophenylboronic acid-block-N-vinyl caprolactam) nanoparticle demonstrated notable glucose sensitivity and effectively reduced blood glucose levels within 72 h. The MTT results indicated that following intraperitoneal administration of nanoparticles at a dosage of 10 mg/kg/day, the cell survival rate exceeded 80%. No adverse effects were observed on the blood biochemistry or the organs, including the heart, liver, spleen, lungs, and kidneys of the mice. These findings suggested that the nanoparticles possess low toxicity to both cellular structures and animal models [120].

Glucose-responsive insulin delivery systems hold significant promise for the management of hyperglycemia in diabetes. A novel biodegradable and charge-switchable plant-derived nanoparticle has been developed. It incorporated glucose-sensitive PBA alongside amine groups. This nanoparticle demonstrated the capability to efficiently form complexes with insulin, resulting in the creation of nanocomplexes [121]. These findings indicated that natural and biodegradable nanoparticles exhibiting glucose responsiveness possess considerable potential for advancement in the domain of insulin delivery systems.

However, clinical studies have shown that an incomplete understanding of quantitative differences between different species may complicate the prediction of clinical outcomes when glucose-responsive insulin is converted to humans. The current lack of data from other clinical trials suggests that there are several major problems with the translation of the currently available glucose-sensitive insulin delivery systems. More research is necessary to assess toxicity and effectiveness [122].

#### 3.4.3. Based on Oral Administration Glucose-Sensitive Drug Delivery System

Compared with injection administration, oral administration has lots of advantages. Oral administration is safe, less painful, and simple to operate, which makes it a better way to administer drugs. Spoken glucose-lowering medication are adequate to subordinate lifeblood sugar position in persons with T2DM. To maximize the effectiveness of oral hypoglycemic drugs, an increasing number of glucose response systems are being developed.

##### Polymers

Smart oral insulin devices have the potential to improve blood glucose management. It reduces the risk of hypoglycemia associated with exogenous insulin therapy while also avoiding many of the drawbacks associated with subcutaneous injections. A responsive nanosystem was developed for intelligent oral insulin. Con A is highly sensitive and specific as a glucose-reactive substance. Fucoidan has hypoglycemic functions and can bind to Con A to form reversible complexes. Under high glucose conditions, free glucose competitively binds to Con A, which causes the nanocarrier to swell and promote insulin release. Furthermore, in the acidic pH environment of the gastrointestinal tract, the positively charged vitamin B12 (VB12) binds strongly to anionic fucoidan. Thereby, it could protect the insulin encapsulated within the carrier. Additionally, VB12 could bind to intestinal epithelial factors to improve the transport rate, thereby promoting the absorption of insulin [84]. Mannose ligand-conjugated nanoparticles were prepared by the shrinkage gel method of insulin and glucose oxidase encapsulated alginate nanocarriers (tripolyphosphate cross-linking). An important finding of glucose oxidase enzyme immobilized alginate chitosan nanoparticles in animal models is that they auto-modulate glycemic response behavior, which could control the reduction of lifeblood glucose. The results recommended that the development of glucose-sensitive nanoparticles might turn out to be a possible policy for spoken insulin delivery [27]. Spoken insulin might become the conventional insulin handing-over method in the future.

However, the management of oral insulin presents significant challenges. It is primarily due to the sensitivity of insulin as a molecule, which is prone to degradation under the prevailing pH conditions of the stomach and intestines. Furthermore, the permeability of intestinal epithelial tissue is notably low, particularly in relation to the substantial volume of insulin. This issue has been solved by encapsulating insulin within mesoporous silica. Mesoporous silica served as a protective barrier. It could preserve the molecular integrity of insulin against the hydrolytic degradation caused by proteins present in the stomach and intestine. Given the high adsorption capacity of mesoporous silica and its biocompatibility, this material exhibited considerable mechanical and chemical resistance, which could make it an ideal candidate for glucose-sensitive controlled release systems. Additionally, these polymers were currently being evaluated for their potential application as biosensors for glucose monitoring [123].

##### Nanoparticles

The glucose-responsive delivery of vitamin K (VK) is investigated by using 3-carboxyphenylboronic acid (CPBA)-functionalized dextran-encapsulated mesoporous silica nanoparticles (MSNs). VK is loaded onto MSN-CPBA by physical adsorption. By forcing CPBA, dextran masks the study of nanoparticles. In the presence of glucose, dextran competes with CPBA, and dextran covers the pores, thereby releasing VK. This would be a promising therapeutic strategy that could improve bioavailability and stimulate responsive delivery of anti-diabetic molecules for the treatment of diabetes [124]. Quercetin nanoparticles (QuNPs) were prepared by polyvinyl alcohol and poly (D,L-lactic acid-hydroxyacetic acid) polymers. Among them, quercetin serves as the base nanocore (QuLNCs). Two layers of PBA were coupled with 3-aminopropyl-triethoxysilane as a functionalizer. Studies of quercetin in vivo have shown that quercetin significantly controlled the release of blood glucose levels [125]. Novel mesoporous silica nanoparticles (MSNs) functionalized by dextro-carboxyphenylboronic acid (CPBA) were reported. This nanoparticle can deliver 1, 25-dihydroxyvitamin D3 (VD) in a glucose-sensitive manner. The MSNs could regulate cellular oxidative stress and inflammation for the treatment of diabetic retinopathy (DR). In human retinal cells supplemented with high glucose, MSN-CPBA increased the bioavailability of VD. It could reduce cellular oxidative stress and inflammation. MSN-CPBA was used as a delivery platform, combined with dextran gating, to provide an effective treatment to improve the bioavailability and efficacy of hydrophobic molecular therapy for DR [126].

Oral medication is one of the tendencies in the care of diabetes. Spoken management of glucose-sensitive drug delivery systems, such as effective handing over of insulin, needs to be further clinically checked for their security, reliability, and effectiveness.

In the early research phase of glucose-responsive drug delivery systems, current research focuses on the application of nanotechnology and biomaterials. Scientists are working to develop new vectors to improve the bioavailability and targeting of drugs. This review summarized clinical studies of glucose-responsive drug delivery systems for the treatment of diabetes (Table 5). However, because there are few clinical trials, relevant data and effectiveness evaluation are still in the exploratory stage. Future drug delivery systems will be optimized to be more suitable for the treatment of diabetes.

### 3.5. Discussion

Advances in glucose response drug delivery continue to evolve. It provides a good opportunity for multiple systems to detect and treat diabetes. As described in this review, the flexible assembly of GOx, PBA, Con A, and others through a variety of synthesis methods is a promising application for the treatment of diabetes. However, despite significant advances in multifaceted research, glucose-sensitive drug delivery systems still present many problems and challenges.

#### 3.5.1. Medication Filling and Susceptibility Topic

At the same time for hypoglycemia, it is very important to ensure sufficient drug loading and appropriate dosage. Therefore, achieving a reasonable and maximum drug loading while maintaining the biological activity of the preloaded drug is one of the research and development goals of researchers. Second, the medication carrier is obligated to answer swiftly to the glucose position for the glucose-responsive drug delivery system. This requires some sensitivity and precision in response to glucose stimuli [127]. However, based on the glucose-sensitive PBA of the chemical reaction, other endogenous particles in the physique are extremely sensitive in the stimulus–response area. It might also encourage its response and release the preloaded medication [122]. Therefore, it is also a major challenge for carrier materials to reasonably avoid the interference of other biomolecules. A perception of the substance combined will be the chief thought in the early step of the plan. In addition, it is necessary to continuously try in vitro and in vivo, rehearse, and simulate various potential interferences.

#### 3.5.2. Material Properties and Other Issues

For the hydrogels that are widely used in current research, it is necessary to have adequate automatic might and sticking to make it difficult to shatter and drop during the drawing process. However, the automatic might of many kinds of hydrogels is not adequate. Due to insufficient mechanical properties, polylactic acid might cause structural rupture under high load conditions and affect the stability of drug release [128]. Polyvinyl alcohol is brittle at low temperatures or high humidity, which might cause failure [129]. The elastic modulus of the material is too high, which limits its adaptability and flexibility in living organisms [130]. Enhancing the automatic honesty and stability of carrier substances is a hot topic in flow research. It is the key to developing potential materials such as hydrogels into carrier materials.

#### 3.5.3. Biocompatibility Subject

Biocompatibility is an extremely important requirement as a carrier of sustained-release drug applications for patients. It needs a medication carrier that could come or go in the physique and become a small, safe particle through hydrolysis or enzymatic hydrolysis. Then, it can be excreted or absorbed through metabolism. It is even kept outside the body to avoid causing a burden. Therefore, the ideal drug carrier should be free of serious toxicity and long-term adverse reactions [131]. For example, although the PBA is widely used, its biocompatibility and safety have not been clinically verified. Polymethyl methacrylate might trigger an immune system response. This could lead to inflammation or allergic reactions [132]. Some biodegradable materials release byproducts during degradation that may be toxic. COx catalyzes the oxidation of glucose to gluconic acid and H_2_O_2_, the latter of which causes enlargement and even serious situation ruin [133]. In response to this dare, research should center on biodegradable and biocompatible substances. The biocompatibility of the material will be enhanced by chemical modification technology. This will improve the ability of cells to attach and proliferate while reducing stimulation of surrounding tissues.

#### 3.5.4. Safety and Reliability Issues

There are some deficiencies in the safety of drug delivery systems, which might affect their effectiveness in clinical applications. Polyurethane releases harmful substances into the body, which could lead to cytotoxicity and tissue damage [134]. Research and development should give priority to materials with low toxicity. Such as gelatin or chitosan these materials are relatively safe in the body [135,136]. In addition, in the procedure of checking the effectiveness of the glucose-sensitive system, the research was continued at the position in vitro and diabetic mice. It has a certain gap with a variety of emergencies, glucose levels, and response effectiveness in clinical practice [137]. In vivo investigation will provide more truth on the deed of enzymes and proteins to better comprehend the mechanism of glucose-sensitive drug delivery and supply better beneficial belongings for diabetics.

#### 3.5.5. Manufacturing Costs Issues

The development of glucose-responsive drug delivery systems often necessitates the use of high-performance biocompatible materials. These are frequently associated with significant costs. Furthermore, the production of such advanced drug delivery systems typically requires specialized equipment and processes. It leads to substantial investment and maintenance expenses. It is well-established that drug development is long-term, labor-intensive, and expensive. The development of novel drug delivery systems demands considerable resources. Consequently, there is a pressing need to investigate the utilization of more cost-effective biomaterials. Additionally, it is imperative to streamline the preparation processes and optimize the methodologies employed. Finally, interdisciplinary collaboration is essential to facilitate the application of innovative technologies. This approach will ultimately lead to the production of low-energy and effective glucose-responsive drug delivery systems.

## 4. Conclusions

With the constant promotion of discipline and technology, the development likelihood of different drugs and glucose-responsive drug delivery systems have become more convincing in diabetes treatment. These systems are mainly assembled based on many types of glucose-sensitive molecules: GOx, PBA, Con A, and others. It could be committed to providing a more intelligent and precise treatment for diabetic patients. However, there are still many problems in glucose-sensitive drug delivery systems, including material performance, sensitivity, biocompatibility, safety, and reliability. Due to the lack of available evidence, the clinical application effect of the glucose-responsive drug delivery system in this review is not sufficient. Glucose-sensitive drug delivery systems will develop to be mature and novel after research. The future will be more unified and include effective care choices such as customized treatment, versatility, and intelligent connectivity. Hence, the development of glucose-responsive drug delivery systems is expected to bring more convenient and effective treatment methods for diabetic patients. It also makes important contributions to improving diabetes management and preventing complications.

## Figures and Tables

**Table 1:** Terms used in the search strategy.

Electronic Database	Search and Terms
Web of Science PubMed	#1 (“Glucose-responsive” OR “Glucose-sensitive”) AND (“Drug delivery system” OR “Drug delivery” OR “Delivery system”) AND (“Diabetes” OR “Diabetes mellitus”)
	#2 (“Drug” OR “Medicine” OR “Biological drug” OR “Chemical drug”) AND (“Diabetes” OR “Diabetes mellitus”) AND (“Therapy” OR “Treatment” OR “Cure”)
	#3 (“Glucose oxidase”) OR (“Phenylboronic acid”) OR (“Concanavalin A”) AND (“Glucose-responsive” OR “Glucose-sensitive”)
	#4 (“Glucose oxidase” OR “GOx” OR “GOD”) OR (“Phenylboronic acid” OR “PBA”) OR (“Concanavalin A” OR “Con A”) AND (“Percutaneous” OR “Transcutaneous”) OR (“Oral” OR “Spoken”) OR (“Injection” OR “Injectable”)

**Table 4:** The advantages and disadvantages of different glucose-sensitive drug delivery systems.

Glucose-Sensitive Drug Delivery Systems	Advantages	Disadvantages	References
Hydrogels	High water content; fast swelling; soft; viscosity; good biocompatibility; self-healing; controllable physical and chemical properties; can prevent drug degradation failure	High cost; slow biodegradation; poor mechanical properties; biosafety hazard	[90,91]
Microneedles	Avoidance of first-pass effects; good patient compliance; reduced probability of drug enzymatic degradation; prolonged maintenance of blood concentrations; absorption rate stabilization; can reduce or eliminate pain; convenient	Can cause localized skin infections; skin irritation; feeling of discomfort; low drug stability	[92,93,94]
Nanoparticles	Large specific surface area and small size; high surface activity; controllable material properties such as size, morphology, structure, etc.; superior light characteristics; high accuracy and wide application range	Poor stability; prone to agglomeration and oxidation; complex preparation process; Safety issues; high cost of preparation; susceptibility to agglomeration	[32,95]
Polymers	High surface area; good biocompatibility; surface modifiable; high porosity; good water solubility; radially ordered porous	Relatively complex synthesis; not stable enough, prone to aggregation; susceptible to inflammatory reactions in vivo	[96]
Nanozymes	Simple functionalization; high stability; have reasonable price; productive potential	Difficult to regulate the activity; difficult to decompose; the application of in vivo transformation is limited	[97,98]
Layer	Good stability; prolonging the duration of action of the drug; easy to prepare	Low carrying capacity; preparative difficulty	[99]

**Table 5:** Compilation of completed/ongoing clinical studies of glucose-responsive drug delivery systems for the treatment of diabetes. (https://clinicaltrials.gov/, 27 September 2024).

Company and Brand Name	Particle Type/Drug	Clinical Trial ID	Study Design\Status	Outcome
Anterogen Co., Ltd.	Hydrogel	NCT03754465	Interventional, parallel assignment Completed	Complete wound repair within the time frame of 12 weeks
Federico II University	Hydrogel	NCT05661474	Interventional (two arms), parallel, single center, randomized, open-label Completed	Faster reepithelialization at the end of twelve weeks
Magda Bayoumi	Hydrogel	NCT04834245	Interventional, parallel, randomized, prospective Completed	Diabetic foot ulcer decrease rate was higher than convention method of dressing
Cyberjaya University College of Medical Sciences	Hydrogel	NCT03816618	Interventional (clinical trial)/parallel assignment Completed	Improvement in the time of healing and wound closure rate
Carlos E Salas	Hydrogel	NCT03700580	Interventional, randomized, double-blind trial Completed	The healing effect of chronic nerve wound in diabetic foot ulcer
VULM s.r.o.	Hydrogel	NCT06584617	Interventional, prospective, multi-center, randomized, open-label Ongoing	Promote treatment and to expedite chronic diabetic foot ulcer healing
Federico II University	Fitostimoline^®^ Hydrogel Versus Saline Gauze	NCT05661474	Interventional, monocentric, two-arm, open-label, randomized, controlled trial Completed	Better efficacy and safety of patients with diabetic foot ulcers
Acera Surgical, Inc.	Hybrid-Scale Fiber Matrix	NCT04927702	Treatment, randomized Interventional, parallel assignment, single (outcomes assessor) Completed	Decrease in diabetes wound area and be living cellular skin substitute
Eclypia	Microneedles	NCT06035367	Interventional, single group assignment, device feasibility, open label Completed	Continuous photoacoustic signal by Neogly in patients with type I diabetes
Emory University	Microneedles	NCT02682056	Interventional, supportive care, open label, single group assignment Completed	Preferable option for monitoring glucose levels among the diabetic pediatric population
University Medical Center Groningen	Polymers	NCT06094920	Interventional, treatment, non-randomized, crossover assignment, open label Ongoing	Optimization of albuminuria-lowering therapies for individual patients with type 2 diabetes
Chia Tai Tianqing Pharmaceutical Group Co., Ltd.	Polymers	NCT06051565	Interventional, open-label, single-center, single-dose, diagnostic, open label Completed	Cardiovascular contrast- injection in diabetic patients
Aviceda Therapeutics, Inc.	Nanoparticles	NCT06181227	Interventional, treatment, randomized, parallel assignment, double (participant outcomes assessor) Completed	Safety and treatment in participants with diabetic macular edema
Clinica Universidad de Navarra, Universidad de Navarra	Nanoparticles	NCT05560412	Interventional, prevention, randomized, crossover assignment, quadruple (participant care provider investigator outcomes assessor)	Better glycemic control
Applied Biologics, LLC	Layer	NCT06564831	Interventional, treatment, randomized, parallel assignment, open label Ongoing	Treatment of non-healing diabetic foot ulcers

## Data Availability

Data are contained within the article.

## Supplementary Materials

The following supporting information can be downloaded at: https://www.mdpi.com/article/10.3390/pharmaceutics16101343/s1, Table S1: Review articles and patents included in the present study.

## List of Abbreviations

T1D (type 1 diabetes); T2DM (type 2 diabetes); DDS (drug delivery system); GOx (glucose oxidases); PBA (phenylboronic acid); Con A (Concanavalin A); GLP-1 (glucagon-like peptide-1); GIP (glucose-dependent insulin polypeptide); OXM (oxyntomodulin); CCK (cholecystokinin); SGLT-2i (sodium–glucose cotransporter 2 inhibitors); COX-2 (cyclooxygenase 2); PPARγ (peroxisome proliferator activated receptor Gamma); ZIF-8 (Zeolitic imidazole framework-8); STZ (streptozotocin); NO (nitric oxide); ROS (reactive oxygen species); H_2_O_2_ (hydrogen peroxide); L-Arg (L-arginine); CS (Chitosan); CIP (Ciprofloxacin hydrochloride); BGM (blood glucose monitoring); CGM (continuous glucose monitoring); EDC (1-(3-Dimethylaminopropyl)-3-ethylcarbodiimide); NHS (N-hydroxysuccinimide); EDAC (1-(3-Dimethylaminopropyl)-3-ethylcarbodiimide hydrochloride); GelMA (gelatin methacryloyl); CPBA (4-carboxyphenylboronic acid); EGCG ((-)-epigallocatechin-3-gallate); MY (myricetin); HA (hyaluronic acid); HAMA (hyaluronic acid methacrylic acid); HMPC (hyaluronic acid methacrylic acid-PBA/catechin); AFPBA (4-(2-acrylamide ethyl carbamoyl)-3-fluorophenyl boronic acid); FCPBA (3-fluoro-4-carboxyphenylboronic acid); GG (guar gum); VB12 (vitamin B12); VK (vitamin K); CPBA (3-carboxyphenylboronic acid); MSNs (mesoporous silica nanoparticles); QuNPs (quercetin nanoparticles); VD (vitamin D3), DR (diabetic retinopathy).
