# Supplementary material for: Recent Advances in the Drugs and Glucose-Responsive Drug Delivery Systems for the Treatment of Diabetes: A Systematic Review
Source: Pharmaceutics. 2024 Oct 20;16(10):1343. doi: 10.3390/pharmaceutics16101343 (PMC11511183; doi:10.3390/pharmaceutics16101343)
Supplement: Supplementary file 1 [file pharmaceutics-16-01343-s001.zip › pharmaceutics-3193829-supplementary.pdf]

pharmaceutics

MDPI

Systematic Review

Supplementary Materials: Recent Advances in the Drugs and Glucose-Responsive Drug Delivery Systems for the Treatment of Diabetes: A Systematic Review

Junyu Liu, Xudong Yi, Jinrui Zhang, Yiman Yao, Pharkphoom Panichayupakaranant and Haixia Chen

Table S1. Review articles and patents included in the present study.

Reference	Year	Aim	Research	Type
[1]	2022	To introduce the current research status of type 2 diabetes and its complications, illustrate the biogenesis of extracellular vesicles.	Diabetes and its complications	Article
[2]	2022	To investigate the risk of gestational diabetes.	Gestational diabetes	Article
[3]	2024	To review the classes of newer hypoglycemic agents and summarize medications currently in phase 2 and 3 clinical trials.	Diabetes agents	Article
[4]	2023	To review the anatomical factors, biochemical factors, and physiology factors that influence delivering drug via oral route.	Oral drug delivery systems	Article
[5]	2023	To review the use of nanomedicines in drug delivery.	Nano drug delivery system	Article
[6]	2022	To report a novel hydrogel with glucose-responsive hyperglycemia regulation and antioxidant activity for enhanced diabetic wound repair.	Glucose-responsive hydrogel	Article
[7]	2021	To present the PRISMA 2020 27-item checklist, an expanded checklist that details reporting recommendations for each item, the PRISMA 2020 abstract checklist, and the revise flow diagrams for original and updated reviews.	PRISMA guideline	Article
[8]	2012	To use HbA1c in the diagnosis of diabetes according to the WHO guidance 2011.	Diagnosis of diabetes	Article
[9]	2021	To convey the important clinical progress of diabetes and guide the clinical practice.	Guideline of T2DM	Article
[10]	2024	To convey the diagnosis and classification of diabetes.	Diagnosis and classification of diabetes	Article
[11]	2021	To convey the newer drug treatments of T2DM.	Treatment of T2DM	Article

[12]	2023	To review the insulin therapy for the management of diabetes mellitus.	Insulin therapy	Article
[13]	2024	To review the usage of long-acting insulin analogs in patients with T2DM	Insulin therapy for T2DM	Article
[14]	2021	To review recent developments concerning adipocyte-cardiomyocyte communications, and identify the most critical questions that remain to be answered in this field.	Diabetes	Article
[15]	2013	To convey that metformin can also modulate clinical outcomes in patients with cancer and concurrent type 2 diabetes.	Metformin treatment for cancer and T2DM	Article
[16]	2017	To review the research, guidelines and consensus on the safe use range of metformin in the treatment of chronic kidney disease, and provide reference for clinical rational use of metformin.	Metformin treatment for T2DM	Article
[17]	2022	To introduce the tirzepatide.	Tirzepatide	Article
[18]	2023	To evaluate the efficacy and safety of once-weekly icodec vs once-daily insulin degludec in people with insulin-naive type 2 diabetes.	Insulin therapy	Article
[19]	2023	To assess the efficacy and safety of orforglipron versus placebo or dulaglutide in participants with T2DM.	Orforglipron for T2DM	Article
[20]	2023	To convey the controversy and complexity of drug pipeline 2Q23.	Drug delivery	Article
[21]	2020	To discuss the classifications, properties, and response mechanisms of the glucose-responsive compounds, glucose oxidase and concanavalin A and the recent progress of glucose-responsive insulin delivery systems.	Glucose-responsive insulin delivery systems	Article
[22]	2022	To review the applications, sources, and recombinant production of glucose oxidase.	Glucose oxidase	Article
[23]	2017	To cover the recent advances in the developments of glucose oxidase-based glucose-sensitive drug delivery systems and their *in vivo* applications for diabetes treatment.	Glucose oxidase-based glucose-responsive delivery system	Article
[24]	2024	To review the latest advances in glucose-responsive microneedle-based systems for transdermal insulin delivery.	Glucose-responsive insulin delivery systems	Article
[25]	2021	To show a new closed-loop and glucose-responsive system for diabetes treatment.	ZIF-8 for glu-cose-responsive drug delivery	Article
[26]	2021	To develop a glucose-responsive delivery system using encapsulated glucose-responsive, acetylated-dextran nanoparticles in porous alginate microgels.	Glucose-responsive insulin delivery systems	Article

[27]	2022	To work on formulation and characterization of a nanocarrier-based bio-responsive, self-regulated oral insulin drug delivery system.	Oral Glucose-Responsive Nanocarrier System	Article
[28]	2023	To report MNs prepared from polylysine-modified cationized silk fibroin for responsive transdermal insulin delivery.	Glucose-responsive silk fibroin microneedles	Article
[29]	2020	To tune insulin release profiles from polymeric nanoparticles by altering the degree of modification of acid-degradable, activated-dextran polymers.	Glucose-responsive nanoparticles	Article
[30]	2018	To study a new pH-sensitive polypeptide capable of self-assemble into hydrogel	pH-Sensitive hydrogel	Patent
[31]	2024	To show a glucose-responsive antibacterial nano-complex microneedle patch.	Glucose-responsive microneedle	Patent
[32]	2023	To review the characteristics of various glucose oxidase-based nanomaterials developed for glucose biosensing and insulin-responsive release as well as research progress, and also highlight the current challenges and opportunities facing this field.	Glucose-oxidase-based nanocomposites	Article
[33]	2020	To show a new class of covalent organic frameworks-based insulin delivery nanocarriers	Glucose-responsive insulin delivery system	Article
[34]	2020	To show a new glucose-responsive delivery for self-regulated insulin release.	Glucose-responsive insulin delivery system	Article
[35]	2022	To prepare the insulin/Ca3PO4 complex and glucose oxidase /Cu3(PO4)2 complex by coprecipitation method.	Glucose-responsive insulin delivery system	Article
[36]	2022	To mix multiple layer-by-layer-insulin microspheres for glucose-mediated insulin release.	Glucose-responsive insulin delivery system	Article
[37]	2024	To develop a cascade nanoreactor hydrogel with arginine Zinc metal-organic framework and glucose oxidase based on chondroitin sulfate and Pluronic to accelerate diabetic infected wound healing.	Glucose oxidase-based cascade nanoreactor	Article
[38]	2023	To develop microenvironment-responsive self-delivery glucose oxidase @ manganese sulfide nanoparticles for diabetic infection treatment.	Glucose oxidase-based nanoparticles	Article
[39]	2022	To design an injectable, "self-healing", and glucose-responsive multifunctional metal-organic drug-loaded hydrogel for diabetic wound healing.	Glucose-responsive hydrogel	Article
[40]	2020	To describe an easy, fast and simple technique of coating a porous polymer layer on stainless steel microneedles that	Glucose-responsive microneedles	Article

		release insulin in a glucose-responsive fashion.		
[41]	2024	To construct a novel in-situ H2O2-sensitive insulin delivery microneedle array patch for the intelligent management of blood glucose in diabetics.	H2O2-sensitive insulin delivery microneedle	Article
[42]	2023	To engineer an injectable, self-healing and antibacterial hydrogel characterized by glucose-responsive and constant NO release behaviors for diabetic wound management.	Glucose-responsive hydrogel	Article
[43]	2022	To develop a cascade nano enzymatic active material by coating glucose oxidase onto peroxidase-like $Fe_2(MoO_4)_3$.	Glucose oxidase-based nanoparticles	Article
[44]	2023	To propose chitosan/MnO_2-glucose oxidase nanocatalysts for the specific catalytic generation of OH to inhibit tumors and bacteria in a hyperglycemic environment.	Glucose oxidase-based nanocatalysts	Article
[45]	2023	To obtain a bi-functional hybrid nanoflower by guanosine monophosphate and glucose oxidase for promoting antibacterial efficacy.	Glucose oxidase-based nanoparticles	Article
[46]	2024	To propose a novel nano-drug delivery based on endogenous glucose-driven cascade reaction for diabetic wound healing.	Glucose-responsive hydrogel	Article
[47]	2022	To design a multifunctional glucose oxidase and catalase nano enzyme-chitosan hydrogel complex for diabetic wounds	Glucose-responsive hydrogel	Article
[48]	2022	To design a nanoreactor based on metal-nanozyme and glucose oxidase combination for starving and photothermal-enhanced chemodynamic antibacterial therapy	Glucose-responsive nanoreactor	Article
[49]	2015	To design the microneedle-array patches loaded with hypoxia-sensitive vesicles for providing fast glucose-responsive insulin delivery.	Glucose-responsive insulin delivery system	Article
[50]	2022	To develop a glucose-mediated dual-responsive drug delivery system for self-regulated administration.	Glucose-responsive delivery system	Article
[51]	2023	To develop a multifunctional cascade nanoreactor based on Fe-driven carbon nanozymes for synergistic photothermal/chemodynamic antibacterial therapy	Glucose-responsive delivery system	Article
[52]	2023	To design self-tandem bio-heterojunctions empower orthopedic implants with amplified chemo-photodynamic anti-pathogenic therapy and boosted diabetic osseointegration	Glucose-responsive delivery system	Article

[53]	2024	To design an antibacterial nanocomposite-integrated microneedle for infected diabetic wound therapy.	Microneedle	Article
[54]	2017	To develop a novel glucose-responsive insulin delivery device using a painless microneedle-array patch containing insulin-loaded vesicles.	Glucose-responsive insulin delivery system	Article
[55]	2022	To develop a lucose-responsive insulin microneedle patch based on phenylboronic acid for 1 diabetes treatment.	Glucose-responsive insulin delivery system	Article
[56]	2023	To develop a glucose-sensitive microneedle patch based on phenylboronic acid modified chitosan particles and poly (vinyl alcohol) / poly(vinylpyrrolidone) hydrogel for the efficient delivery of insulin.	Glucose-responsive insulin delivery system	Article
[57]	2024	To design dually crosslinked insulin polyionic micelles based on triblock polymers of o-glycol and phenylboronic acid-functionalized poly (ethylene glycol)-poly(dimethylamino carbonate)-poly(dimethylamino-trimethylene carbonate) for sustained glucose-responsive insulin release.	Glucose-responsive insulin delivery system	Article
[58]	2020	To design switch-type glucose-responsive double-layer crosslinked polymer micellar drug delivery system.	Glucose-responsive drug delivery system	Patent
[59]	2024	To design sugar-responsive gel for drug delivery device	Glucose-responsive drug delivery system	Patent
[60]	2020	To develop phenylboronic-acid-functionalized dextran nanoplatforms to enhance the permeability of cargos and boost penetration.	Glucose-responsive insulin delivery system	Article
[61]	2019	To summarize some recent attempts at the developments of PBA-mediated glucose-sensitive gels for self-regulated drug delivery.	Glucose-responsive drug delivery system	Article
[62]	2022	To design a novel glucose-responsive antioxidant hybrid hydrogel for enhanced diabetic wound repair.	Glucose-responsive hybrid	Article
[63]	2022	To modify hyaluronic acid methacrylate with phenylboronic acid and develop a glucose-responsive hyaluronic acid derivative.	Glucose-responsive hydrogel	Article
[64]	2023	To explore the function of folliculin-interacting protein 1 (FNIP1) work to alter mitochondrial morphology.	Glucose-responsive hydrogel	Article
[65]	2023	To present the development of a straightforward fabrication strategy for glucose-responsive protein-polymer hybrid hydrogels.	Glucose-responsive hydrogel	Article

[66]	2023	To report a novel kind of PBA-based glucose-responsive microneedles derived from PBA-based glucose-responsive hydrogels.	Glucose-responsive hydrogel	Article
[67]	2019	To report an artificial insulin delivery system, mimicking physiological basal and prandial insulin secretion to achieve real-time glycemic control and reduce the risk of hypoglycemia.	Glucose-responsive insulin delivery system	Article
[68]	2019	To report a smart microneedle composed of a semi-interpenetrating network hydrogel prepared by biocompatible silk fibroin and phenylboronic acid/acrylamide for glucose-responsive insulin delivery.	Glucose-responsive insulin delivery system	Article
[69]	2022	To design a composite system that can realize the sol-gel phase transition only through simple ambient temperature or glucose concentration stimulation.	Glucose-responsive hydrogel	Article
[70]	2024	To present an innovative method for synthesizing pH-thermo-glucose responsive poly (NIPA-co-DMAEMA)-PBA hydrogel nanoparticles via single-step aqueous free radical polymerization.	Glucose-responsive hydrogel	Article
[71]	2022	To prepare dynamic-covalent hydrogels by a new diboronate motif with enhanced glucose binding and importantly improved glucose specificity-leveraging the ability of rigid diboronates.	Glucose-responsive hydrogel	Article
[72]	2022	To design a thermogel platform prepared from temperature-induced micelles formed into a network by PBA-Diol cross-linking using a formulation-centered approach to maximize glucose-responsive insulin delivery.	Glucose-responsive hydrogel	Article
[73]	2024	To develop a self-healing, dual-layer, drug-carrying microneedle for diabetic wound healing.	Glucose-responsive microneedle	Article
[74]	2023	To present a glucose-responsive smart hydrogel platform based on phenylboronic acid-functionalized natural silk fibroin protein for regulated insulin delivery.	Glucose-responsive hydrogel	Article
[75]	2018	To prepare novel cellulose/phenylboronic acid composite intelligent bio-hydrogel and its glucose, pH-responsive behaviors.	Glucose-responsive hydrogel	Article
[76]	2019	To present new insights into material design and physicochemical interactions that are relevant for the use of glucose-responsive polymeric hydrogels in continuously operating biosensor systems.	Glucose-responsive hydrogel	Article

[77]	2024	To review recent insights into glucose-responsive Concanavalin A-based smart hydrogels for controlled insulin delivery.	Glucose-responsive hydrogel	Article
[78]	2023	To prepare the proposed flexible enzyme-free glucose sensor by combining Concanavalin A-based glucose-responsive hydrogels with green-synthetic silver particles on laser direct-writing graphene electrodes.	Glucose-responsive hydrogel	Article
[79]	2022	To propose a capacitive glucose sensor with high linearity and a wide detection range based on a glucose-responsive DexG-Con A hydrogel.	Glucose-responsive hydrogel	Article
[80]	2022	To design demand regulation of blood glucose level by biocompatible oxidized starch-Con A nanogels for glucose-responsive release of exenatide.	Glucose-responsive hydrogel	Article
[81]	2023	To focus on employing a thermoresponsive co-forming matrix between Pluronic F-127 (PL) and structurally robust chitosan via EDC/NHS coupling	Glucose-responsive hydrogel	Article
[82]	2024	To fabricate concanavalin A conjugated phthalocyanine-loaded cochleates as a glucose-sensitive lipidic system and estimate its efficacy in streptozotocin-induced male Sprague Dawley diabetic rats for 28 days.	Glucose-responsive drug delivery system	Article
[83]	2022	To develop a targeted oral delivery system for diabetes.	Glucose-responsive drug delivery system	Article
[84]	2024	To develop a responsive nanosystem vitamin B12Fucoidan-Concanavalin A with anti-inflammatory action for smart oral delivery of insulin.	Glucose-responsive insulin delivery system	Article
[85]	2018	To design medicine-loaded glucose-sensitive orally available nanoparticles useful for preparing medicine for treating diabetes.	Glucose-responsive drug delivery system	Patent
[86]	2022	To report a glucose-responsive insulin delivery microneedle array patch that is loaded with red blood cell vesicles or liposome nanoparticles containing glucose transporters bound with glucosamine-modified insulin.	Glucose-responsive insulin delivery system	Article
[87]	2023	To report biodegradable, partially oxidized alginate carriers for glucose-responsive nanoparticles or islet cells.	Glucose-responsive nanoparticles	Article
[88]	2020	To optimize the synthetic route of synthetic-Lectin.	Glucose-responsive hydrogel	Article
[89]	2021	To develop linkers with low levels of spontaneous hydrolysis but increase level	Glucose-responsive hydrogel	Article

		of hydrolysis with rising concentrations of glucose.		
[90]	2023	To start from the dynamic bonds that are used to realize the self-healing properties of biopolymer-based hydrogels, and discuss their advantages and disadvantages.	Glucose-responsive hydrogel	Article
[91]	2023	To review the preparation methods of dynamic reversible covalent self-healing hydrogels at home and abroad and their application research progress in drug transportation, wound dressing, tissue engineering and biosensors.	Glucose-responsive hydrogel	Article
[92]	2023	To introduce the advantages and disadvantages of some microneedles and nano-formulations.	Glucose-responsive microneedle	Article
[93]	2023	To introduce the types of materials of the microneedles firstly.	Glucose-responsive microneedle	Article
[94]	2022	To review research progress on traditional Chinese medicine percutaneous microneedle preparation.	Glucose-responsive microneedle	Article
[95]	2020	To discuss different types of nanoparticles for skin permeation enhancement and targeted delivery to skin organelles.	Glucose-responsive nanoparticles	Article
[96]	2022	To describe a brief and simple explanation of each method, along with some recent results and each technique's advantages and disadvantages.	Polymers	Article
[97]	2021	To introduce the recent progress of carbon-based nanozymes including carbon nanozymes, heteroatom-doped carbon nanozymes and metal-doped carbon nanozymes.	Nanozymes	Article
[98]	2024	To review advanced enzyme-mimicking theragnostic tool of nanozymes.	Nanozymes	Article
[99]	2017	To review research progress of Layer-by-layer self -assembly technology in preparation of thin films.	Layer-by-layer thin films	Article
[100]	2023	To review progress in glucose-sensitive hydrogels for biomedical applications.	Glucose-sensitive hydrogel	Article
[101]	2022	To review efficiency of multifunctional antibacterial hydrogels for chronic wound healing in diabetes.	Hydrogel	Article
[102]	2024	To explore treatment modalities for wounds in individuals with diabetes.	Glucose-sensitive hydrogel	Article
[103]	2023	To introduce reversible boronic bonds to create an intelligent antioxidant hydrogel scaffold.	Glucose-sensitive hydrogel	Article
[104]	2018	To introduce the method of obtaining glucose-sensitive polymer hydrogels that can be used as carriers for the controlled	Glucose-sensitive hydrogel	Patent

		release of insulin upon the appearance of glucose.		
[105]	2024	To construct insulin-loaded phenyl-borate-ester-cross-linked microneedles with the polyzwitterion property based on the modified epsilon-polylysine and poly-(vinyl alcohol).	Glucose-sensitive microneedles	Article
[106]	2023	To design a novel self-crosslinkable and glucose-responsive polymer-based microneedle patch to deliver insulin at hyperglycemia.	Glucose-sensitive microneedles	Article
[107]	2022	To develop a glucose-responsive insulin-releasing hydrogel for microneedle dressing fabrication and then investigate its effects on diabetic wound healing.	Glucose-sensitive hydrogel	Article
[108]	2022	To develop glucose response insulin micro-needle patch comprises a micro-needle body comprising framework carrier, glucose-sensitive factor and insulin gluconate	Glucose-sensitive microneedles	Patent
[109]	2024	To design a glucose-responsive complex useful in preparing medicine for treating diabetes.	Glucose-responsive drug delivery system	Patent
[110]	2020	To design a smart glucose-sensitive insulin delivery platform based on transcutaneous microneedles.	Glucose-responsive drug delivery system	Article
[111]	2021	To design a blood glucose measurement system using glucose-reactive fluorescence characteristic	Glucose-responsive measurement system	Patent
[112]	2023	To discusses oral administration of insulin, glucose-responsive devices and innovative administration routes.	Glucose-responsive drug delivery system	Article
[113]	2023	To prepare a glucose-responsive self-healing hydrogel based on polysaccharides.	Glucose-sensitive hydrogel	Article
[114]	2015	To develop a pyrene-containing phenylboronic acid functionalized low-molecular-weight hydrogelator to develop glucose-sensitive insulin release.	Glucose-sensitive hydrogel	Article
[115]	2023	To design Chitosan-based injectable hydrogels and fabricat through the dynamic crosslinking of dualreversible covalent bonds for precise insulin release.	Glucose-sensitive hydrogel	Article
[116]	2023	To review advancements and applications of injectable hydrogel composites in biomedical research and therapy.	Glucose-sensitive hydrogel	Article
[117]	2022	To design glycopolymer nanoparticles with on-demand glucose-responsive insulin delivery and low-hypoglycemia risks for type 1 diabetic treatment.	Glucose-responsive insulin delivery system	Article

[118]	2019	To develop polymer microneedles integrated with glucose-responsive mesoporous bioactive glass nanoparticles for transdermal delivery of insulin for self-regulate and painless transdermal administration.	Glucose-responsive insulin delivery system	Article
[119]	2016	To design a new glucose-sensitive porous microsphere/polymer composite gel useful for developing insulin implanted gel for long-acting sustained release medicine preparation.	Glucose-responsive insulin delivery system	Patent
[120]	2019	To prepare insulin-loaded poly (3-acrylamidophenylboronic acid-block-N-vinyl caprolactam) p(AAPBA-b-NVCL) nanoparticles for effectively lowering the blood sugar levels within 72 hrs.	Glucose-responsive insulin delivery system	Article
[121]	2023	To engineer the biodegradable and charge-switchable phytoglycogen nanoparticles capable of glucose-stimulated insulin release.	Glucose-responsive insulin delivery system	Article
[122]	2020	To review the glucose-responsive insulin and delivery systems.	Glucose-responsive insulin delivery system	Article
[123]	2018	To review the applications of mesoporous silica in biosensing and controlled release of insulin.	Glucose-responsive insulin delivery system	Article
[124]	2022	To examine the glucose-responsive delivery of vitamin K by using dextran-capped mesoporous silica nanoparticles (MSNs) functionalized with 3-carboxyphenylboronic acid.	Glucose-responsive drug delivery system	Article
[125]	2024	To demonstrate a controlled release of Quercetin from formulation of quercetin -based layered nanocarriers with higher stability and anti-diabetic activity.	Glucose-responsive drug delivery system	Article
[126]	2024	To study the efficacy of dextran-gated carboxyphenylboronic acid-functionalized mesoporous silica nanoparticles for glucose-sensitive delivery of 1,25-dihydroxyvitamin D3 to modulate cellular oxidative stress and inflammation for managing diabetic retinopathy.	Glucose-responsive drug delivery system	Article
[127]	2020	To focus on stimuli-responsive, reservoir-based insulin delivery devices.	Glucose-responsive insulin delivery system	Article
[128]	2024	To review the effects of nucleating agents and processing on the crystallization and mechanical properties of polylactic acid.	Glucose-responsive drug delivery system	Article
[129]	2024	To review the utilization of novel basalt fiber pellets from micro- to macro-scale, and from basic to applied fields.	Glucose-responsive drug delivery system	Article

[130]	2023	To reveal how experimental sound velocities can improve the accuracy of common thermal conductivity models and present a critical discussion of Gruneisen parameter estimates from elastic moduli.	Elastic moduli	Article
[131]	2021	To review the application of boronate bond in drug delivery systems.	Drug delivery system	Article
[132]	2023	To review the different modification methods of poly methyl methacrylate bone cement for orthopedic surgery applications.	Poly methyl methacrylate	Article
[133]	2016	To review the glucose-sensitive polymer nanoparticles for self-regulated drug delivery.	Glucose-responsive drug delivery system	Article
[134]	2023	To review recent advances in functional polyurethane chemistry.	Polyurethane	Article
[135]	2023	To review chitosan hydrogel as tissue engineering scaffolds for vascular regeneration applications.	Chitosan hydrogel	Article
[136]	2023	To review the application of gelatin microspheres in bone tissue engineering.	Gelatin microspheres	Article
[137]	2021	To review the progress of intelligent-responsive insulin delivery mediated by glucose oxidase.	Glucose-responsive insulin delivery system	Article

1. Zhang, M.; Wang, L.; Chen, Z. Research progress of extracellular vesicles in type 2 diabetes and its complications. *Diabetic Medicine* **2022**, *39*, doi:10.1111/dme.14865.
2. Kragelund Nielsen, K.; Davidsen, E.; Husted Henriksen, A.; Andersen, G.S. Gestational Diabetes and International Migration. *Journal of the Endocrine Society* **2022**, *7*, doi:10.1210/jendso/bvac160.
3. Villasenor, M.; Selzer, A.R. Preoperative Patient Evaluation: Newer Hypoglycemic Agents. *Anesthesiology clinics* **2024**, *42*, 41-52, doi:10.1016/j.anclin.2023.08.004.
4. Lou, J.; Duan, H.; Qin, Q.; Teng, Z.; Gan, F.; Zhou, X.; Zhou, X. Advances in Oral Drug Delivery Systems: Challenges and Opportunities. *Pharmaceutics* **2023**, *15*, doi:10.3390/pharmaceutics15020484.
5. Prakash, S. Nano-based drug delivery system for therapeutics: a comprehensive review. *Biomedical Physics & Engineering Express* **2023**, *9*, doi:10.1088/2057-1976/acedb2.
6. Xu, Z.; Liu, G.; Li, Q.; Wu, J. A novel hydrogel with glucose-responsive hyperglycemia regulation and antioxidant activity for enhanced diabetic wound repair. *Nano Research* **2022**, *15*, 5305-5315, doi:10.1007/s12274-022-4192-y.
7. Page, M.J.; McKenzie, J.E.; Bossuyt, P.M.; Boutron, I.; Hoffmann, T.C.; Mulrow, C.D.; Shamseer, L.; Tetzlaff, J.M.; Akl, E.A.; Brennan, S.E.; et al. The PRISMA 2020 statement: an updated guideline for reporting systematic reviews. *Bmj-British Medical Journal* **2021**, *372*, doi:10.1136/bmj.n71.
8. John, W.G.; Diabet, U.K.D.H.A.C. Use of HbA1c in the diagnosis of diabetes mellitus in the UK. The implementation of World Health Organization guidance 2011. *Diabetic Medicine* **2012**, *29*, 1350-1357, doi:10.1111/j.1464-5491.2012.03762.x.
9. Chinese Diabetes, S. Guideline for the prevention and treatment of type 2 diabetes mellitus in China(2020 edition)(Part 1). *Chinese Journal of Practical Internal Medicine* **2021**, *41*, 668-695.

10. ElSayed, N.A.; Aleppo, G.; Bannuru, R.R.; Bruemmer, D.; Collins, B.S.; Ekhlaspour, L.; Gaglia, J.L.; Hilliard, M.E.; Johnson, E.L.; Khunti, K.; et al. 2. Diagnosis and Classification of Diabetes: <i>Standards of Care in Diabetes-2024</i>. *Diabetes Care* **2024**, *47*, S20-S42, doi:10.2337/dc24-S002.
11. Guo, J.; Smith, S.M. Newer drug treatments for type 2 diabetes Guidance developed in partnership with patients recommends a risk based approach. *Bmj-British Medical Journal* **2021**, *373*, doi:10.1136/bmj.n1171.
12. Nkonge, K.M.; Nkonge, D.K.; Nkonge, T.N. Insulin Therapy for the Management of Diabetes Mellitus: A Narrative Review of Innovative Treatment Strategies. *Diabetes Therapy* **2023**, *14*, 1801-1831, doi:10.1007/s13300-023-01468-4.
13. Aktas, G.; Taslamacioglu Duman, T. Current usage of long-acting insulin analogs in patients with type 2 diabetes mellitus. *Expert Review of Endocrinology & Metabolism* **2024**, *19*, 155-161, doi:10.1080/17446651.2024.2320631.
14. Wang, Y.; Lau, W.B.; Ma, X. "Know Diabetes by Heart": role of adipocyte-cardiomyocyte communications. *Medical review (2021)* **2021**, *1*, 14-17, doi:10.1515/mr-2021-0008.
15. Yin, M.; Zhou, J.; Gorak, E.J.; Quddus, F. Metformin Is Associated With Survival Benefit in Cancer Patients With Concurrent Type 2 Diabetes: A Systematic Review and Meta-Analysis. *Oncologist* **2013**, *18*, 1248-1255, doi:10.1634/theoncologist.2013-0111.
16. Zhang, Y.; Kuang, H. Metformin in treatment of type 2 diabetes mellitus complicated with chronic kidney disease:research advances. *Journal of International Pharmaceutical Research* **2017**, *44*, 306-310.
17. Syed, Y.Y. Tirzepatide: First Approval. *Drugs* **2022**, *82*, 1213-1220, doi:10.1007/s40265-022-01746-8.
18. Lingvay, I.; Asong, M.; Desouza, C.; Gourdy, P.; Kar, S.; Vianna, A.; Vilsboll, T.; Vinther, S.; Mu, Y. Once-Weekly Insulin Icodec vs Once-Daily Insulin Degludec in Adults With Insulin-Naive Type 2 Diabetes The ONWARDS 3 Randomized Clinical Trial. *Jama-Journal of the American Medical Association* **2023**, *330*, 228-237, doi:10.1001/jama.2023.11313.
19. Frias, J.P.; Hsia, S.; Eyde, S.; Liu, R.; Ma, X.; Konig, M.; Kazda, C.; Mather, K.J.; Haupt, A.; Pratt, E.; et al. Efficacy and safety of oral orforglipron in patients with type 2 diabetes: a multicentre, randomised, dose-response, phase 2 study. *Lancet* **2023**, *402*, 472-483, doi:10.1016/s0140-6736(23)01302-8.
20. Hodgson, J. Drug pipeline 2Q23-controversy and complexity. *Nature Biotechnology* **2023**, *41*, 1041-1043, doi:10.1038/s41587-023-01886-6.
21. Shen, D.; Yu, H.; Wang, L.; Khan, A.; Haq, F.; Chen, X.; Huang, Q.; Teng, L. Recent progress in design and preparation of glucose-responsive insulin delivery systems. *Journal of Controlled Release* **2020**, *321*, 236-258, doi:10.1016/j.jconrel.2020.02.014.
22. Khatami, S.H.; Vakili, O.; Ahmadi, N.; Soltani Fard, E.; Mousavi, P.; Khalvati, B.; Maleksabet, A.; Savardashtaki, A.; Taheri-Anganeh, M.; Movahedpour, A. Glucose oxidase: Applications, sources, and recombinant production. *Biotechnology and Applied Biochemistry* **2022**, *69*, 939-950, doi:10.1002/bab.2165.
23. Zhao, L.; Wang, L.; Zhang, Y.; Xiao, S.; Bi, F.; Zhao, J.; Gai, G.; Ding, J. Glucose Oxidase-Based Glucose-Sensitive Drug Delivery for Diabetes Treatment. *Polymers* **2017**, *9*, doi:10.3390/polym9070255.
24. Martinez-Navarrete, M.; Cordeiro, A.S.; Perez-Lopez, A.; Guillot, A.J.; Melero, A.; Aparicio-Blanco, J. Latest advances in glucose-responsive microneedle-based systems for transdermal insulin delivery. *International Journal of Biological Macromolecules* **2024**, *263*, doi:10.1016/j.ijbiomac.2024.130301.
25. Yin, Z.; Lin, M.; Xu, Y.; Wang, Z.; Cai, Y.; Yang, X. Enzyme and Au nanoparticles encapsulated ZIF-8 for glucose responsive closed-loop drug delivery. *Materials Letters* **2021**, *301*, doi:10.1016/j.matlet.2021.130276.
26. Volpatti, L.R.; Facklam, A.L.; Cortinas, A.B.; Lu, Y.-C.; Matranga, M.A.; MacIsaac, C.; Hill, M.C.; Langer, R.; Anderson, D.G. Microgel encapsulated nanoparticles for glucose-responsive insulin delivery. *Biomaterials* **2021**, *267*, doi:10.1016/j.biomaterials.2020.120458.
27. Maurya, R.; Ramteke, S.; Guru, P.; Jain, N.K. Oral Glucose-Responsive Nanocarrier System for Management of Diabetes. *Journal of Endocrinology and Metabolism* **2022**, *12*, 146-160, doi:10.14740/jem747.
28. Tan, G.; Jiang, F.; Jia, T.; Qi, Z.; Xing, T.; Kundu, S.C.; Lu, S. Glucose-Responsive Silk Fibroin Microneedles for Transdermal Delivery of Insulin. *Biomimetics* **2023**, *8*, doi:10.3390/biomimetics8010050.

29. Volpatti, L.R.; Matranga, M.A.; Cortinas, A.B.; Delcassian, D.; Daniel, K.B.; Langer, R.; Anderson, D.G. Glucose-Responsive Nanoparticles for Rapid and Extended Self-Regulated Insulin Delivery. *Acs Nano* **2020**, *14*, 488-497, doi:10.1021/acsnano.9b06395.

30. Huang, W.; Qian, H.; Dai, Y.; Fu, M.; Li, X.; Zhang, C. New pH-sensitive polypeptide capable of self-assemble into hydrogel useful as polypeptide material for constructing glucose responsive insulin release system, and drug for treating diabetes, comprises specific amino acid sequences. CN107529533-A; CN107529533-B.

31. Ling, G.; Zhou, Q.; Zhang, P. Glucose-responsive antibacterial nano-complex microneedle patch used for e.g. treating bacterially infected diabetic wounds, comprises nano-complex ciprofloxacin hydrochloride/glucose oxidase-zeolitic imidazolate framework. CN117643568-A.

32. Yang, D.; Cai, C.; Liu, K.; Peng, Z.; Yan, C.; Xi, J.; Xie, F.; Li, X. Recent advances in glucose-oxidase-based nanocomposites for diabetes diagnosis and treatment. *Journal of Materials Chemistry B* **2023**, *11*, 7582-7608, doi:10.1039/d3tb01097j.

33. Zhang, G.; Ji, Y.; Li, X.; Wang, X.; Song, M.; Gou, H.; Gao, S.; Jia, X. Polymer-Covalent Organic Frameworks Composites for Glucose and pH Dual-Responsive Insulin Delivery in Mice. *Advanced Healthcare Materials* **2020**, *9*, doi:10.1002/adhm.202000221.

34. Zhang, C.; Hong, S.; Liu, M.-D.; Yu, W.-Y.; Zhang, M.-K.; Zhang, L.; Zeng, X.; Zhang, X.-Z. pH-sensitive MOF integrated with glucose oxidase for glucose-responsive insulin delivery. *Journal of Controlled Release* **2020**, *320*, 159-167, doi:10.1016/j.jconrel.2020.01.038.

35. Xiao, Y.; Wang, X.; Xie, D. Application of hyaluronic acid microneedles in insulin intelligent delivery system for the treatment of diabetes. *Sheng wu gong cheng xue bao = Chinese journal of biotechnology* **2022**, *38*, 3433-3442, doi:10.13345/j.cjb.220129.

36. Yang, Y.; Wang, X.; Yuan, X.; Zhu, Q.; Chen, S.; Xia, D. Glucose-activatable insulin delivery with charge-conversional polyelectrolyte multilayers for diabetes care. *Frontiers in Bioengineering and Biotechnology* **2022**, *10*, doi:10.3389/fbioe.2022.996763.

37. Xiang, G.; Wang, B.; Zhang, W.; Dong, Y.; Tao, J.; Zhang, A.; Chen, R.; Jiang, T.; Zhao, X. A Zn-MOF-GOx-based cascade nanoreactor promotes diabetic infected wound healing by NO release and microenvironment regulation. *Acta Biomaterialia* **2024**, *182*, 245-259, doi:10.1016/j.actbio.2024.05.015.

38. Ge, Y.; Rong, F.; Lu, Y.; Wang, Z.; Liu, J.; Xu, F.; Chen, J.; Li, W.; Wang, Y. Glucose Oxidase Driven Hydrogen Sulfide-Releasing Nanocascade for Diabetic Infection Treatment. *Nano Letters* **2023**, *23*, 6610-6618, doi:10.1021/acs.nanolett.3c01771.

39. Yang, J.; Zeng, W.; Xu, P.; Fu, X.; Yu, X.; Chen, L.; Leng, F.; Yu, C.; Yang, Z. Glucose-responsive multifunctional metal-organic drug-loaded hydrogel for diabetic wound healing. *Acta Biomaterialia* **2022**, *140*, 206-218, doi:10.1016/j.actbio.2021.11.043.

40. Ullah, A.; Choi, H.J.; Jang, M.; An, S.; Kim, G.M. Smart Microneedles with Porous Polymer Layer for Glucose-Responsive Insulin Delivery. *Pharmaceutics* **2020**, *12*, doi:10.3390/pharmaceutics12070606.

41. Kuang, Y.; Xue, F.; Dai, Z.; Zhu, Y.; Liu, Q.; Chen, H. Anti-inflammatory PEGylated bilirubin microneedle patch for diabetes treatment. *Applied Materials Today* **2024**, *39*, doi:10.1016/j.apmt.2024.102295.

42. Zhou, X.; Zhao, B.; Wang, L.; Yang, L.; Chen, H.; Chen, W.; Qiao, H.; Qian, H. A glucose-responsive nitric oxide release hydrogel for infected diabetic wounds treatment. *Journal of Controlled Release* **2023**, *359*, 147-160, doi:10.1016/j.jconrel.2023.05.047.

43. Zhang, Y.; Li, D.; Xu, Y.; Niu, Y. Application of a Cascaded Nanozyme in Infected Wound Recovery of Diabetic Mice. *Acs Biomaterials Science & Engineering* **2022**, *8*, 1522-1531, doi:10.1021/acsbiomaterials.1c01590.

44. Degradable nanocatalyst enables antitumor/antibacterial therapy and promotion of wound healing for diabetes via self-enhanced cascading reaction. *Chinese Chemical Letters* **2023**, *34*.

45. Tang, Q.; Shi, L.; Yang, B.; Liu, W.; Li, B.; Jin, Y. A biomineralized bi-functional hybrid nanoflower to effectively combat bacteria <i>via</i> a glucose-powered cascade catalytic reaction. *Journal of Materials Chemistry B* **2023**, *11*, 3413-3421, doi:10.1039/d2tb02704f.

46. Zhang, J.; Li, W.; Tao, Z.; Zhou, X.; Chen, X.; Zhou, J.; Sun, H.; Fang, Y.; Liu, Y. Endogenous glucose-driven cascade reaction of nano-drug delivery for boosting multidrug-resistant bacteria-infected diabetic wound healing. *Journal of Colloid and Interface Science* **2024**, *672*, 63-74, doi:10.1016/j.jcis.2024.05.204.

47. Li, Z.; Fan, X.; Luo, Z.; Loh, X.J.; Ma, Y.; Ye, E.; Wu, Y.-L.; He, C.; Li, Z. Nanoenzyme-chitosan hydrogel complex with cascade catalytic and self-reinforced antibacterial performance for accelerated healing of diabetic wounds. *Nanoscale* **2022**, *14*, 14970-14983, doi:10.1039/d2nr04171e.

48. Wang, T.; Chen, T.; Zhu, J.; Wang, S.; Wen, W.; Zhang, X.; Tang, H.; Liang, J.; Wang, S.; Xiong, H.; et al. Acidity-responsive cascade nanoreactor based on metal-nanozyme and glucose oxidase combination for starving and photothermal-enhanced chemodynamic antibacterial therapy. *Chemical Engineering Journal* **2022**, *446*, doi:10.1016/j.cej.2022.137172.
49. Yu, J.; Zhang, Y.; Ye, Y.; DiSanto, R.; Sun, W.; Ranson, D.; Ligler, F.S.; Buse, J.B.; Gu, Z. Microneedle-array patches loaded with hypoxia-sensitive vesicles provide fast glucose-responsive insulin delivery. *Proceedings of the National Academy of Sciences of the United States of America* **2015**, *112*, 8260-8265, doi:10.1073/pnas.1505405112.
50. Wang, Y.; Cheng, S.; Fan, W.; Jiang, Y.; Yang, J.; Tong, Z.; Jiang, G. Dual responsive block copolymer coated hollow mesoporous silica nanoparticles for glucose-mediated transcutaneous drug delivery. *Chinese Journal of Chemical Engineering* **2022**, *51*, 35-42, doi:10.1016/j.cjche.2021.07.019.
51. Shen, Y.; Nie, C.; Pan, T.; Zhang, W.; Yang, H.; Ye, Y.; Wang, X. A multifunctional cascade nanoreactor based on Fe-driven carbon nanozymes for synergistic photothermal/chemodynamic antibacterial therapy. *Acta Biomaterialia* **2023**, *168*, 580-592, doi:10.1016/j.actbio.2023.07.006.
52. Shu, R.; Sun, J.; Li, B.; Gao, X.; He, M.; Chan, Y.K.; Shi, J.; Bai, D.; Yang, W.; Deng, Y. Self-Tandem Bio-Heterojunctions Empower Orthopedic Implants with Amplified Chemo-Photodynamic Anti-Pathogenic Therapy and Boosted Diabetic Osseointegration. *Advanced Functional Materials* **2023**, *33*, doi:10.1002/adfm.202214873.
53. Zhou, Q.; Li, X.; Gao, N.; Ling, G.; Zhang, P. A multimodal therapy for infected diabetic wounds based on glucose-responsive nanocomposite-integrated microneedles. *Journal of Materials Chemistry B* **2024**, *12*, 1007-1021, doi:10.1039/d3tb02609d.
54. Yu, J.; Qian, C.; Zhang, Y.; Cui, Z.; Zhu, Y.; Shen, Q.; Ligler, F.S.; Buse, J.B.; Gu, Z. Hypoxia and H₂O₂ Dual-Sensitive Vesicles for Enhanced Glucose-Responsive Insulin Delivery. *Nano Letters* **2017**, *17*, 733-739, doi:10.1021/acs.nanolett.6b03848.
55. Zong, Q.; Zhou, R.; Zhao, Z.; Wang, Y.; Liu, C.; Zhang, P. Glucose-responsive insulin microneedle patch based on phenylboronic acid for 1 diabetes treatment. *European Polymer Journal* **2022**, *173*, doi:10.1016/j.eurpolymj.2022.111217.
56. Wang, Y.; Yu, H.; Wang, L.; Hu, J.; Feng, J.; Shen, D.; Hong, Y.; Liu, J.; Chen, D. Microneedles with Two-Stage Glucose-Sensitive Controlled Release for Long-Term Insulin Delivery. *Acs Biomaterials Science & Engineering* **2023**, *9*, 2534-2544, doi:10.1021/acsbiomaterials.3c00137.
57. Ma, Y.; Xing, Y.; Han, F.; Xu, J.; Qian, H.; Chen, W.; Huang, D. Dually crosslinked degradable polyionic micelles for sustained glucose-responsive insulin release. *Biomaterials Science* **2024**, *12*, 3202-3211, doi:10.1039/d4bm00314d.
58. Chen, W.; Xing, Y.; Qian, H.; Huang, D.; Yu, Y. Switch-type glucose-responsive double-layer crosslinked polymer micellar drug delivery system used for preparing drug, is prepared by performing ring-opening polymerization of methoxy polyethylene glycol, and mixing and self assembling. CN111658783-A; CN111658783-B.
59. Michiko, I.; Hiroko, M.; Sayaka, K.; Akira, M.; Matsumoto, R.; Kanai, S.; Matsumoto, Y.; Ito, M. Sugar-responsive gel for drug delivery device, comprises polymerization reaction product of mixture containing gelling agent containing N-isopropylmethacrylamide and N,N-diethylacrylamide, phenylboronic acid monomer, hydroxyl-based monomer, crosslinking agent and photopolymerization initiator. WO2024158039-A1; JP2024106274-A.
60. Wei, X.; Duan, X.; Zhang, Y.; Ma, Z.; Li, C.; Zhang, X. Internalization Mechanism of Phenylboronic-Acid-Decorated Nanoplatform for Enhanced Nasal Insulin Delivery. *Acs Applied Bio Materials* **2020**, *3*, 2132-2139, doi:10.1021/acsabm.0c00002.
61. Wang, C.; Lin, B.; Zhu, H.; Bi, F.; Xiao, S.; Wang, L.; Gai, G.; Zhao, L. Recent Advances in Phenylboronic Acid-Based Gels with Potential for Self-Regulated Drug Delivery. *Molecules* **2019**, *24*, doi:10.3390/molecules24061089.
62. Xu, Z.; Liu, G.; Huang, J.; Wu, J. Novel Glucose-Responsive Antioxidant Hybrid Hydrogel for Enhanced Diabetic Wound Repair. *Acs Applied Materials & Interfaces* **2022**, *14*, 7680-7689, doi:10.1021/acsami.1c23461.
63. Xu, Z.; Liu, G.; Liu, P.; Hu, Y.; Chen, Y.; Fang, Y.; Sun, G.; Huang, H.; Wu, J. Hyaluronic acid-based glucose-responsive antioxidant hydrogel platform for enhanced diabetic wound repair. *Acta Biomaterialia* **2022**, *147*, 147-157, doi:10.1016/j.actbio.2022.05.047.

64. Zhang, W.; Zha, K.; Xiong, Y.; Hu, W.; Chen, L.; Lin, Z.; Yu, C.; Zhou, W.; Cao, F.; Hu, H.; et al. Glucose-responsive, antioxidative HA-PBA-FA/EN106 hydrogel enhanced diabetic wound healing through modulation of FEM1b-FNIP1 axis and promoting angiogenesis. *Bioactive Materials* **2023**, *30*, 29-45, doi:10.1016/j.bioactmat.2023.07.006.
65. Ali, A.; Saroj, S.; Saha, S.; Rakshit, T.; Pal, S. In Situ-Forming Protein-Polymer Hydrogel for Glucose-Responsive Insulin Release. *Acs Applied Bio Materials* **2023**, doi:10.1021/acsabm.2c00951.
66. Lu, Y.; Yu, H.; Wang, L.; Shen, D.; Liu, J. Preparation of phenylboronic acid-based glucose-responsive hydrogels and microneedles for regulated delivery of insulin. *European Polymer Journal* **2023**, *192*, doi:10.1016/j.eurpolymj.2023.112061.
67. Lv, J.; Wu, G.; Liu, Y.; Li, C.; Huang, F.; Zhang, Y.; Liu, J.; An, Y.; Ma, R.; Shi, L. Injectable dual glucose-responsive hydrogel-micelle composite for mimicking physiological basal and prandial insulin delivery. *Science China-Chemistry* **2019**, *62*, 637-648, doi:10.1007/s11426-018-9419-3.
68. Chen, S.; Matsumoto, H.; Moro-oka, Y.; Tanaka, M.; Miyahara, Y.; Suganami, T.; Matsumoto, A. Smart Microneedle Fabricated with Silk Fibroin Combined Semi interpenetrating Network Hydrogel for Glucose-Responsive Insulin Delivery. *Acs Biomaterials Science & Engineering* **2019**, *5*, 5781-5789, doi:10.1021/acsbiomaterials.9b00532.
69. Hu, D.; Ju, X.; Xie, R.; Wang, W.; Liu, Z.; Chu, L. Injectable Temperature- and Glucose-responsive Composite Hydrogels for Controlled Release of Insulin at Physiological pH. *Materials Review* **2022**, *36*.
70. Chafran, L.; Carfagno, A. Synthesis of multi-responsive poly(NIPA-<i>co</i>-DMAEMA)-PBA hydrogel nanoparticles in aqueous solution for application as glucose-sensitive insulin-releasing nanoparticles. *Journal of Diabetes and Metabolic Disorders* **2024**, doi:10.1007/s40200-024-01421-7.
71. Xiang, Y.; Xian, S.; Ollier, R.C.; Yu, S.; Su, B.; Pramudya, I.; Webber, M.J. Diboronate crosslinking: Introducing glucose specificity in glucose-responsive dynamic-covalent networks. *Journal of Controlled Release* **2022**, *348*, 601-611, doi:10.1016/j.jconrel.2022.06.016.
72. Xian, S.; VandenBerg, M.A.; Xiang, Y.; Yu, S.; Webber, M.J. Glucose-Responsive Injectable Thermogels via Dynamic-Covalent Cross-Linking of Pluronic Micelles. *Acs Biomaterials Science & Engineering* **2022**, doi:10.1021/acsbiomaterials.2c00979.
73. Liu, M.; You, J.; Zhang, Y.; Zhang, L.; Quni, S.; Wang, H.; Zhou, Y. Glucose-Responsive Self-Healing Bilayer Drug Microneedles Promote Diabetic Wound Healing Via a Trojan-Horse Strategy. *Acs Applied Materials & Interfaces* **2024**, *16*, 24351-24371, doi:10.1021/acsami.4c03050.
74. Maity, B.; Moorthy, H.; Govindaraju, T. Glucose-Responsive Self-Regulated Injectable Silk Fibroin Hydrogel for Controlled Insulin Delivery. *Acs Applied Materials & Interfaces* **2023**, *15*, 49953-49963, doi:10.1021/acsami.3c07060.
75. Peng, H.; Ning, X.; Wei, G.; Wang, S.; Dai, G.; Ju, A. The preparations of novel cellulose/phenylboronic acid composite intelligent bio-hydrogel and its glucose, pH-responsive behaviors. *Carbohydrate Polymers* **2018**, *195*, 349-355, doi:10.1016/j.carbpol.2018.04.119.
76. Walter, S.V.; Ennen-Roth, F.; Buening, D.; Denizer, D.; Ulbricht, M. Glucose-Responsive Polymeric Hydrogel Materials: From a Novel Technique for the Measurement of Glucose Binding toward Swelling Pressure Sensor Applications. *Acs Applied Bio Materials* **2019**, *2*, 2464-2480, doi:10.1021/acsabm.9b00168.
77. Bercea, M.; Lupu, A. Recent Insights into Glucose-Responsive Concanavalin A-Based Smart Hydrogels for Controlled Insulin Delivery. *Gels* **2024**, *10*, doi:10.3390/gels10040260.
78. Hu, Y.; Yang, D.; Zhang, H.; Gao, Y.; Zhang, W.; Yin, R. A High-Linearity Glucose Sensor Based on Silver-Doped Con A Hydrogel and Laser Direct Writing. *Polymers* **2023**, *15*, doi:10.3390/polym15061423.
79. Yin, R.; Xin, J.; Yang, D.; Gao, Y.; Zhang, H.; Qian, Z.; Zhang, W. High-Linearity Hydrogel-Based Capacitive Sensor Based on <i>Con A</i>-Sugar Affinity and Low-Melting-Point Metal. *Polymers* **2022**, *14*, doi:10.3390/polym14204302.
80. On demand regulation of blood glucose level by biocompatible oxidized starch-Con A nanogels for glucose-responsive release of exenatide. *Journal of Controlled Release* **2022**, *352*, 673-684, doi:10.1016/j.jconrel.2022.10.039.
81. Mansoor, S.; Adeyemi, S.A.; Kondiah, P.P.D.; Choonara, Y.E. A Closed Loop Stimuli-Responsive Concanavalin A-Loaded Chitosan-Pluronic Hydrogel for Glucose-Responsive Delivery of Short-Acting Insulin Prototyped in RIN-5F Pancreatic Cells. *Biomedicines* **2023**, *11*, doi:10.3390/biomedicines11092545.

82. Govardhane, S.; Shende, P. Phthalocyanine-based glucose-responsive nanocochleates for dynamic prevention of β-cell damage in diabetes. *Journal of Liposome Research* **2024**, *34*, 44-59, doi:10.1080/08982104.2023.2209642.
83. Xu, M.; Huang, J.; Jiang, S.; He, J.; Wang, Z.; Qin, H.; Guan, Y.-Q. Glucose sensitive konjac glucomannan/concanavalin A nanoparticles as oral insulin delivery system. *International Journal of Biological Macromolecules* **2022**, *202*, 296-308, doi:10.1016/j.ijbiomac.2022.01.048.
84. Zhou, J.; Ma, H.; Guan, M.; Feng, J.; Dong, X.; Wei, Y.; Zhang, T. Anti-inflammatory Fucoidan-ConA oral insulin nanosystems for smart blood glucose regulation. *International Journal of Pharmaceutics* **2024**, *659*, doi:10.1016/j.ijpharm.2024.124250.
85. Guan, Y.; Zhang, L.; Qiu, J.; Xu, M. Medicine-loaded glucose-sensitive orally available nanoparticle useful for e.g. preparing medicine for treating diabetes comprises nanoparticles reversibly cross-linked by glucomannan, concanavalin A and crosslinking agent. CN108721605-A; CN108721605-B.
86. Chen, Q.; Xiao, Z.; Wang, C.; Chen, G.; Zhang, Y.; Zhang, X.; Han, X.; Wang, J.; Ye, X.; Prausnitz, M.R.; et al. Microneedle Patches Loaded with Nanovesicles for Glucose Transporter- Mediated Insulin Delivery. *Acs Nano* **2022**, *16*, 18223-18231, doi:10.1021/acsnano.2c05687.
87. Volpatti, L.R.; Bochenek, M.A.; Facklam, A.L.; Burns, D.M.; MacIsaac, C.; Morgart, A.; Walters, B.; Langer, R.; Anderson, D.G. Partially Oxidized Alginate as a Biodegradable Carrier for Glucose-Responsive Insulin Delivery and Islet Cell Replacement Therapy. *Advanced Healthcare Materials* **2023**, *12*, doi:10.1002/adhm.202201822.
88. Lan, R.-y.; Zhu, L.; Wang, X.-f.; Wu, W.-t. Synthesis and Glucose-responsiveness of Synthetic-Lectin-contained Microgels. *Acta Polymerica Sinica* **2020**, *51*, 961-968, doi:10.11777/j.issn1000-3304.2020.20128.
89. Mannerstedt, K.; Mishra, N.K.; Engholm, E.; Lundh, M.; Madsen, C.S.; Pedersen, P.J.; Le-Huu, P.; Pedersen, S.L.; Buch-Manson, N.; Borgstrom, B.; et al. An Aldehyde Responsive, Cleavable Linker for Glucose Responsive Insulins. *Chemistry-a European Journal* **2021**, *27*, 3166-3176, doi:10.1002/chem.202004878.
90. Wang, X.; Zhang, H.J.; Yang, Y.; Chen, Y.; Zhu, X.; You, X. Biopolymer-based self-healing hydrogels: A short review. *Giant* **2023**, *16*, doi:10.1016/j.giant.2023.100188.
91. Xu, H.; Shen, Y.; Pan, H.; Xu, L. Progress in preparation and application of self-healing hydrogels based on dynamic reversible covalent bonds. *New Chemical Materials* **2023**, *51*, 87-92.
92. Li, H.; Lu, Z.; Gan, T.; Jian, W.; Feng, J.; Jin, Y.; Tu, L. Research progress of nano-formulation combined with microneedles for transdermal drug delivery. *Chinese Journal New Drugs* **2023**, *32*, 1089-1098.
93. Luo, X.; Yang, L.; Cui, Y. Microneedles: materials, fabrication, and biomedical applications. *Biomedical Microdevices* **2023**, *25*, doi:10.1007/s10544-023-00658-y.
94. Zhao, Y.; Ning, H.; Zhang, Y.; Li, X.; Li, M.; Li, Y.; Hou, W.; Wang, Y. Research progress on traditional Chinese medicine percutaneous microneedle preparation. *Chinese Traditional and Herbal Drugs* **2022**, *53*, 2550-2559.
95. Ghasemiyeh, P.; Mohammadi-Samani, S. Potential of Nanoparticles as Permeation Enhancers and Targeted Delivery Options for Skin: Advantages and Disadvantages. *Drug Design Development and Therapy* **2020**, *14*, 3271-3289, doi:10.2147/dddt.S264648.
96. do Amaral Montanheiro, T.L.; Schatkoski, V.M.; Canuto de Menezes, B.R.; Pereira, R.M.; Ribas, R.G.; Martinez de Freitas, A.d.S.; Lemes, A.P.; Vaz Fernandes, M.H.F.; Thim, G.P. Recent progress on polymer scaffolds production: Methods, main results, advantages and disadvantages. *Express Polymer Letters* **2022**, *16*, 197-219, doi:10.3144/expresspolymlett.2022.16.
97. Chen, Y.-F.; Zhang, Y.; Jiao, L.; Yan, H.-Y.; Gu, W.-L.; Zhu, C.-Z. Research Progress of Carbon-based Nanozymes for Biosensing. *Chinese Journal of Analytical Chemistry* **2021**, *49*, 907-921, doi:10.19756/j.issn.0253-3820.211258.
98. Pant, G.; Singh, S.; Choudhary, P.K.; Ramamurthy, P.C.; Singh, H.; Garlapati, D.; Singh, J.; Kumar, G.; Khan, N.A.; Zahmatkesh, S. Nanozymes: advance enzyme-mimicking theragnostic tool: a review. *Clean Technologies and Environmental Policy* **2024**, doi:10.1007/s10098-023-02716-8.
99. Duan, T.; Zheng, W.; Liu, X.; Huang, Y.; Wang, D.; Wang, J.; Guo, Y.; Li, N.; Wang, L.; Xin, P. Research Progress of Layer-by-layer Self -assembly Technology in Preparation of Thin Films. *Engineering Plastics Application* **2017**, *45*, 139-142.

100. Li, Y.; Feng, G.; Liu, J.; Yang, T.; Hou, R.; Liu, J.; Wang, X. Progress in Glucose-Sensitive Hydrogels for Biomedical Applications. *Macromolecular Chemistry and Physics* **2023**, *224*, doi:10.1002/macp.202300257.
101. Ji, J.-Y.; Ren, D.-Y.; Weng, Y.-Z. Efficiency of Multifunctional Antibacterial Hydrogels for Chronic Wound Healing in Diabetes: A Comprehensive Review. *International Journal of Nanomedicine* **2022**, *17*, 3163-3176, doi:10.2147/ijn.S363827.
102. Li, X.; Bai, L.; Zhang, X.; Fang, Q.; Chen, G.; Xu, G. Application of <i>Bletilla striata</i> polysaccharide hydrogel for wound healing among in diabetes. *Colloids and Surfaces B-Biointerfaces* **2024**, *241*, doi:10.1016/j.colsurfb.2024.114033.
103. Chen, F.; Qin, J.; Wu, P.; Gao, W.; Sun, G. Glucose-Responsive Antioxidant Hydrogel Accelerates Diabetic Wound Healing. *Advanced Healthcare Materials* **2023**, *12*, doi:10.1002/adhm.202300074.
104. Valuev, L.I.; Valuev, I.L.; Vanchugova, L.V. Method of obtaining glucose-sensitive polymer hydrogels that can be used as carriers for controlled release of insulin upon the appearance of glucose. RU2652126-C1.
105. Shen, D.; Yu, H.; Wang, L.; Wang, Y.; Feng, J.; Li, C. Electrostatic-Interaction-Aided Microneedle Patch for Enhanced Glucose-Responsive Insulin Delivery and Three-Meal-Per-Day Blood-Glucose Regulation. *Acs Applied Materials & Interfaces* **2024**, *16*, 4449-4461, doi:10.1021/acsami.3c16540.
106. Liu, J.F.; Ghavaminejad, A.; Lu, B.; Mirzaie, S.; Samarikhalaj, M.; Giacca, A.; Wu, X.Y. "Smart" Matrix Microneedle Patch Made of Self-Crosslinkable and Multifunctional Polymers for Delivering Insulin On-Demand. *Advanced Science* **2023**, *10*, doi:10.1002/advs.202303665.
107. Guo, Z.; Liu, H.; Shi, Z.; Lin, L.; Li, Y.; Wang, M.; Pan, G.; Lei, Y.; Xue, L. Responsive hydrogel-based microneedle dressing for diabetic wound healing. *Journal of Materials Chemistry B* **2022**, *10*, 3501-3511, doi:10.1039/d2tb00126h.
108. Zong, Q.; Zhang, P. Glucose response insulin micro-needle patch comprises micro-needle body comprising framework carrier, glucose sensitive factor and insulin gluconate, and substrate, which is phenylboronic acid or phenylboronic acid derivative-modified methacrylated hyaluronic acid, or methacrylated hyaluronic acid. CN114601918-A; CN114601918-B.
109. Gu, Z.; Wang, J.; Zhang, J. Glucose-responsive complex useful in preparing medicine for treating diabetes comprises mutually complexed phenylboronic acid-based polylysine and insulin with diol structure, where complexing force include e.g. electrostatic attraction. WO2024067600-A1; CN117771387-A.
110. Lin, Y.; Hu, W.; Bai, X.; Ju, Y.; Cao, C.; Zou, S.; Tong, Z.; Cen, C.; Jiang, G.; Kong, X. Glucose- and pH-Responsive Supramolecular Polymer Vesicles Based on Host-Guest Interaction for Transcutaneous Delivery of Insulin. *Acs Applied Bio Materials* **2020**, *3*, 6376-6383, doi:10.1021/acsabm.0c00813.
111. Kim, J.H.; Lee, M.; Lim, H.S.; Choi, H.S. Blood glucose measurement system using glucose-reactive fluorescence characteristic, has glucose-responsive fluorescent unit which is attached to skin of user in form of patch and change fluorescence according to glucose in body fluid. KR2021023199-A; KR2348087-B1.
112. Marinheiro, D.; Martel, F.; Ferreira, B.J.M.L.; Daniel-da-Silva, A.L.L. Silica-Based Nanomaterials for Diabetes Mellitus Treatment. *Bioengineering-Basel* **2023**, *10*, doi:10.3390/bioengineering10010040.
113. Tan, W.-w.; Lei, S.-s.; Long, T.; Xu, Z.-l.; Li, D.-f.; Mu, C.-d.; Ge, L.-m. Polysaccharide Based Injectable Self-healing Hydrogels with Glucose Responsive Drug Release Behavior. *Acta Polymerica Sinica* **2023**, *54*, 1155-1165, doi:10.11777/j.issn1000-3304.2023.23024.
114. Mandal, D.; Mandal, S.K.; Ghosh, M.; Das, P.K. Phenylboronic Acid Appended Pyrene-Based Low-Molecular-Weight Injectable Hydrogel: Glucose-Stimulated Insulin Release. *Chemistry-a European Journal* **2015**, *21*, 12042-12052, doi:10.1002/chem.201501170.
115. Zhang, J.; Chen, F.; Yu, D.; Liang, Z.; Dai, F.; Liang, H.; Li, H.; Tan, H.; Zhao, L. Chitosan-based injectable hydrogels with dual glucose sensors for precise control of insulin release and diabetes mellitus therapy. *International Journal of Pharmaceutics* **2023**, *643*, doi:10.1016/j.ijpharm.2023.123246.
116. Omidian, H.; Chowdhury, S.D. Advancements and Applications of Injectable Hydrogel Composites in Biomedical Research and Therapy. *Gels* **2023**, *9*, doi:10.3390/gels9070533.

117. Fu, Y.; Sun, Y.; Chen, M.; Xing, W.; Xu, Y.; Qian, X.; Zhu, W. Glycopolymer Nanoparticles with On-Demand Glucose-Responsive Insulin Delivery and Low-Hypoglycemia Risks for Type 1 Diabetic Treatment. *Biomacromolecules* **2022**, *23*, 1251-1258, doi:10.1021/acs.biomac.1c01496.

118. Jiang, G.; Xu, B.; Zhu, J.; Zhang, Y.; Liu, T.; Song, G. Polymer microneedles integrated with glucose-responsive mesoporous bioactive glass nanoparticles for transdermal delivery of insulin. *Biomedical Physics & Engineering Express* **2019**, *5*, doi:10.1088/2057-1976/ab3202.

119. Zhang, J.; Yao, D.; Guo, R.; Dong, A. New glucose-sensitive porous microsphere/polymer composite gel useful for developing insulin implanted gel for long-acting sustained release medicine preparation. CN106038478-A; CN106038478-B.

120. Wu, J.-z.; Yang, Y.; Li, S.; Shi, A.; Song, B.; Niu, S.; Chen, W.; Yao, Z. Glucose-Sensitive Nanoparticles Based On Poly(3-Acrylamidophenylboronic Acid-Block-N-Vinylcaprolactam) For Insulin Delivery. *International Journal of Nanomedicine* **2019**, *14*, 8059-8072, doi:10.2147/ijn.S220936.

121. Xu, R.; Bhangu, S.K.; Sourris, K.C.; Vanni, D.; Sani, M.-A.; Karas, J.A.; Alt, K.; Niego, B.e.; Ale, A.; Besford, Q.A.; et al. An Engineered Nanosugar Enables Rapid and Sustained Glucose-Responsive Insulin Delivery in Diabetic Mice. *Advanced Materials* **2023**, *35*, doi:10.1002/adma.202210392.

122. Wang, J.; Wang, Z.; Yu, J.; Kahkoska, A.R.; Buse, J.B.; Gu, Z. Glucose-Responsive Insulin and Delivery Systems: Innovation and Translation. *Advanced Materials* **2020**, *32*, doi:10.1002/adma.201902004.

123. Soenmez, M.; Ficai, D.; Ficai, A.; Alexandrescu, L.; Georgescu, M.; Trusca, R.; Gurau, D.; Titu, M.A.; Andronescu, E. Applications of mesoporous silica in biosensing and controlled release of insulin. *International Journal of Pharmaceutics* **2018**, *549*, 179-200, doi:10.1016/j.ijpharm.2018.07.037.

124. Sarkar, S.; Sadhukhan, P.; Das, D.; Basyach, P.; Das, J.; Das, M.R.; Saikia, L.; Wann, S.B.; Kalita, J.; Sil, P.C.; et al. Glucose-Sensitive Delivery of Vitamin K by Using Surface-Functionalized, Dextran-Capped Mesoporous Silica Nanoparticles To Alleviate Hyperglycemia. *Acs Applied Materials & Interfaces* **2022**, *14*, 26489-26500, doi:10.1021/acsami.2c05974.

125. Shah, P.; Shende, P. Engineering of Layered Nanocarriers of Quercetin for the Treatment of Diabetes Using Box-Behnken Design. *Particle & Particle Systems Characterization* **2024**, *41*, doi:10.1002/ppsc.202300037.

126. Sarkar, S.; Osman, N.; Thrimawithana, T.; Wann, S.B.; Kalita, J.; Manna, P. Alleviation of Diabetic Retinopathy by Glucose-Triggered Delivery of Vitamin D via Dextran-Gated Functionalized Mesoporous Silica Nanoparticles. *Acs Applied Bio Materials* **2024**, *7*, 1260-1270, doi:10.1021/acsabm.3c01200.

127. Fuchs, S.; Shariati, K.; Ma, M. Stimuli-Responsive Insulin Delivery Devices. *Pharmaceutical Research* **2020**, *37*, doi:10.1007/s11095-020-02918-5.

128. Gao, P.; Masato, D. The Effects of Nucleating Agents and Processing on the Crystallization and Mechanical Properties of Polylactic Acid: A Review. *Micromachines* **2024**, *15*, doi:10.3390/mi15060776.

129. Ahmed, T.; Bediwy, A.; Azzam, A.; Elhadary, R.; El-Salakawy, E.; Bassuoni, M.T. Utilization of Novel Basalt Fiber Pellets from Micro- to Macro-Scale, and from Basic to Applied Fields: A Review on Recent Contributions. *Fibers* **2024**, *12*, doi:10.3390/fib12020017.

130. Isotta, E.; Peng, W.; Balodhi, A.; Zevalkink, A. Elastic Moduli: a Tool for Understanding Chemical Bonding and Thermal Transport in Thermoelectric Materials. *Angewandte Chemie-International Edition* **2023**, doi:10.1002/anie.202213649.

131. Wang, L.; Sheng, X.; Wang, J.; Zhang, Y. Application of Boronate Bond in Drug Delivery System. *Chinese Journal of Organic Chemistry* **2021**, *41*, 567-581, doi:10.6023/cjoc202006060.

132. Ghasemi, F.; Jahani, A.; Moradi, A.; Ebrahimzadeh, M.H.; Jirofti, N. Different Modification Methods of Poly Methyl Methacrylate (PMMA) Bone Cement for Orthopedic Surgery Applications. *Archives of Bone and Joint Surgery-Abjs* **2023**, *11*, 485-492, doi:10.22038/abjs.2023.71289.3330.

133. Zhao, L.; Xiao, C.; Wang, L.; Gai, G.; Ding, J. Glucose-sensitive polymer nanoparticles for self-regulated drug delivery. *Chemical Communications* **2016**, *52*, 7633-7652, doi:10.1039/c6cc02202b.

134. Jiang, R.; Zheng, X.; Zhu, S.; Li, W.; Zhang, H.; Liu, Z.; Zhou, X. Recent Advances in Functional Polyurethane Chemistry: From Structural Design to Applications. *Chemistryselect* **2023**, *8*, doi:10.1002/slct.202204132.
135. Wang, Q.; Wang, X.; Feng, Y. Chitosan Hydrogel as Tissue Engineering Scaffolds for Vascular Regeneration Applications. *Gels* **2023**, *9*, doi:10.3390/gels9050373.
136. Zhai, Y.; Li, J.; Abuduaini, A.; Yang, Z.; Yu, Z.; Chen, Y.; Liu, H.; Rong, X. Application of gelatin microspheres in bone tissue engineering. *Sheng wu gong cheng xue bao = Chinese journal of biotechnology* **2023**, *39*, 3724-3737, doi:10.13345/j.cjb.220871.
137. Yang, J.; Ma, Y.; Huang, D.; Qian, H.; Chen, W. Progress of intelligent-responsive insulin delivery mediated by glucose oxidase. *Journal of China Pharmaceutical University* **2021**, *52*, 663-674.
